# Hormonal contraception and the risk of suicidal behaviour: a Swedish nationwide register-based study

**DOI:** 10.1136/bmjopen-2025-105694

**Published:** 2025-11-27

**Authors:** Jurate Aleknaviciute, Donghao Lu, Vide Gotby, Emma M Frans, Ralf Kuja-Halkola, Hedvig Engberg, Henning Tiemeier, Paul Lichtenstein, Fang Fang, Steven A. Kushner, Zheng Chang

**Affiliations:** 1 Department of Psychiatry, Erasmus Medical Center, Rotterdam, Zuid-Holland, Netherlands; 2 Department of Medical Epidemiology and Biostatistics, Karolinska Institutet, Stockholm, Sweden; 3 Department of Women's and Children's Health, Karolinska Institutet, Stockholm, Sweden; 4 Department of Gynecology and Reproductive Medicine, Karolinska University Hospital, Stockholm, Sweden; 5 Department of Social and Behavioral Sciences, Harvard T.H. Chan School of Public Health, Boston, MA, USA; 6 Unit of Integrative Epidemiology, Institute of Environmental Medicine, Karolinska Institutet, Stockholm, Sweden; 7 Department of Psychiatry, Vagelos College of Physicians & Surgeons, Columbia University, New York, NY, USA; 8 Stavros Niarchos Foundation (SNF) Center for Precision Psychiatry & Mental Health, Columbia University, New York, NY, USA

**Keywords:** Suicide & self-harm, MENTAL HEALTH, Adolescent, PUBLIC HEALTH

## Abstract

**ABSTRACT:**

**Objectives:**

To determine whether hormonal contraceptives are associated with subsequent risks of suicidal behaviour and depression among women of reproductive age.

**Design:**

Nationwide register-based study.

**Setting:**

Swedish national population using health and death registers. Nationwide registries provided individual-level information about the use of hormonal contraception, suicidal behaviour, depression and potential confounders.

**Participants:**

All women in Sweden from 1 January 2006 to 31 December 2013.

**Outcomes measures:**

Suicidal behaviour events or registered deaths due to suicide were identified through the National Patient Register and Cause of Death Register, respectively. Clinical diagnoses of depression were obtained from the patient register. Cox regression models were used to estimate HRs with 95% CIs of suicidal behaviour and depression in women using hormonal contraceptives.

**Results:**

We followed more than two million women for a median of 6.8 years (12.4 million person-years in total). No increased risk was observed among women using oral contraceptives or non-oral combined oestrogen/progestin formulations. Non-oral progestin-only contraceptives were associated with an increased risk of suicidal behaviour using both population-based (HR=1.17, 95% CI 1.13 to 1.21) and within-individual (HR=1.16, 95% CI 1.11 to 1.21) analyses. Age-stratified analyses revealed that during late adolescence (age 15–18), use of oral contraceptives or non-oral combined formulations was associated with an increased risk of suicidal behaviour (range of HRs: 1.09–1.35), an effect that was not observed in adulthood. In contrast, non-oral progestin-only contraceptives were associated with an increased risk of suicidal behaviour during both late adolescence and adulthood.

**Conclusions:**

We found no overall increased risk of suicidal behaviour among women using oral contraceptives or non-oral combined formulations. However, the observed increased risk associated with hormonal contraceptive use during adolescence, as well as with non-oral progestin-only contraception—particularly gonane-containing formulations—across the entire reproductive window warrants attention and further investigation.

**STRENGTHS AND LIMITATIONS OF THIS STUDY**

- Nationwide sample of more than two million women.
- Longitudinal cohort design allowing within-individual comparisons.
- Objective measures of exposures and outcomes.
- Reliance on prescription data for hormonal contraceptive use has been well-validated, but is still a proxy metric.
- Information regarding less severe suicidal behaviour that did not require medical care was incomplete.

## Introduction

 Hundreds of millions of women use hormonal contraceptives worldwide, providing a convenient and effective method of family planning.^1^ Among women of reproductive age, the point prevalence of hormonal contraceptive use is nearly 60% in the United States and most European countries.^1^ Given their widespread use, the risk of adverse events is of significant public health concern. The thromboembolic risk of hormonal contraceptives containing oestrogens has been extensively documented, particularly among women who smoke or are of older age.^2 3^ Consequently, clinical guidelines have been adapted to require that women are properly informed about the associated risk of thromboembolism prior to initiating hormonal contraceptives.^4^

There is currently no consensus on the potential adverse effects of hormonal contraceptive use broadly on mental health.^5^,^17^ Several large cohort studies have found positive associations between hormonal contraceptive use and increased risks of depression and suicide,^12 13^ supported by a systematic review and meta-analysis,^15^ and a nationally representative study reporting elevated suicidality among hormonal contraceptive users.^14^ An additional cohort study among young women observed increased suicidal behaviour, though this risk declined with longer duration of use.^16^ In contrast, other observational studies have not demonstrated significant associations between oral contraceptive use and depression, and evidence linking hormonal contraceptive use to suicidality remains limited.^7^,^10^ A recent large cohort study also found no significant association between hormonal contraceptive use and the risk of attempted suicide.¹⁷

Notably, the existing literature on hormonal contraception and mental health. Outcomes have predominantly focused on younger women,^12^,^16^ ^18^ with several studies reporting significant associations between hormonal contraceptive use and suicidal behaviour within this population.^13 14 16^ Given the partial overlap in risk factors for suicidality between adolescents and adults,^19^ it remains an important area of investigation whether the association between hormonal contraception and suicidal behaviour persists into adulthood. From a public health perspective, this is also a compelling issue given the substantial proportion of middle-aged women using hormonal contraception for family planning,^1 20^ among whom the suicide rate is much higher than young women.^21^ Moreover, although a wide range of different types of hormonal contraception is available, there has been an increasing preference for long-lasting reversible forms of contraceptives, for which only limited data is available regarding non-oral hormonal contraceptive use and potential adverse effects on mental health outcomes.^6 12 13 22^

Given the ongoing discussion and sometimes conflicting findings surrounding hormonal contraception and mental health, this Swedish nationwide study examined the relationship between distinct hormonal contraceptive formulations and the risk of suicidal behaviour across the entire window of reproductive ages, using both population-wide and within-individual analyses. Considering that the majority of middle-aged women are either prior or current users of hormonal contraception, we also assessed the risk of suicidal behaviour among women following discontinuation of hormonal contraception compared with those who continued. Furthermore, given that depression is among the strongest predictors of suicidal behaviour, we quantified the association between hormonal contraceptive use and depression diagnosis.

## Methods

### Study population

We conducted a population-based cohort study using Swedish national registers. Based on the Total Population Register, we identified 2 232 774 women who were born in Sweden between 1956 and 1998. Using the unique personal identification number assigned to all residents in Sweden,^23^ we linked on the Causes of Death and Migration Registers for follow-up, beginning on 1 January 2006 or at age 15, whichever came later, until age 50, death, emigration or 31 December 2013, whichever occurred first. We therefore excluded women who died (n=35 748) or emigrated (n=1 56 977) before cohort entry. Moreover, we excluded follow-up time between the onset of pregnancy until 6 months postpartum. We obtained the date of childbirth from the Medical Birth Register and estimated the date of conception according to documentation of gestational age. We obtained socioeconomic information from the Longitudinal Integration Database for Health Insurance and Labour Market Studies (LISA). The final cohort included 2 039 988 women, including a subsample of 475 196 (23.3%) living in Stockholm County during the study period, for sensitivity analysis (see Appendix 1 for details).

This study was approved by the Regional Ethics Committee at Karolinska Institutet (Stockholm, Sweden); 2013/862-31/5.

### Use of hormonal contraception

We obtained information on hormonal contraceptives by dispensed prescriptions recorded in the Swedish Prescribed Drug Register. Information is available since July 2005 regarding Anatomical Therapeutic Chemical (ATC) codes, dispensation and dosage for prescription medications in all pharmacies in Sweden.^24^ Using ATC codes, hormonal contraceptives were sub-categorised by route of administration (oral or non-oral formulation) and hormonal class (oestrogen/progestin combination or progestin-only) (online supplemental table S1).

For each woman, the use of hormonal contraception was defined as a time-varying exposure, from the day of dispensation until the end of the prescription period, which was estimated according to the total amount of dispensed medication and the Defined Daily Dose (DDD, online supplemental table S2). We assumed a gap between two consecutive same-type prescriptions of up to 4 weeks as a continuous use, whereas a gap beyond that was regarded as a discontinuation. Use of a given hormonal contraceptive was truncated on dispensation of another contraceptive or pregnancy. Considering our aim to investigate the association between the use of hormonal contraception and subsequent suicidal behaviour across the entire window of reproductive age, we used non-exposed periods as the reference condition, with the underlying assumptions of immediate transient effects of hormonal contraceptives, the absence of carry over, a constant effect across continuous use periods, and no depletion of susceptibility.

### Outcomes

We defined a suicidal behaviour event as a suicide attempt or death by suicide. Consistent with previous research, suicide attempt was defined as intentional self-harm (ICD-10: X60-X84) or self-harm of undetermined intent (ICD-10: Y10-Y34) in the National Patient Register.^25^,^27^ ^28^ Depression was considered a secondary outcome, operationally defined based on a clinical diagnosis of ICD-10 codes F32–F33 recorded in the National Patient Register, with diagnostic data available from 1997 onwards. History of psychiatric disorders (ICD F10-F99) and suicidal behaviour was defined by the corresponding diagnoses any time before the start of follow-up from the National Patient Register.

The Patient Register includes nationwide information on hospital discharge records since 1987 and outpatient visits to speciality care since 2001. However, it is important to note that information on cases arising from primary care visits is largely missing. In contrast, the Causes of Death Register covers over 99% of deaths occurring in Sweden.

## Covariables

Civil partnership and parity were treated as time-varying variables across follow-up periods. Civil partnership was defined as either married or in a legally registered partnership. Parity data were extracted from the Medical Birth Register and Multi-Generation Register. History of psychiatric disorders (ICD F10-F99) and suicidal behaviour was obtained from the National Patient Register. Mean individualised household income was binned into quartiles throughout the follow-up period. The highest level of educational attainment during follow-up was represented categorically as primary school, high school, college or unknown.

## Statistical analysis

### Descriptive statistics

We compared demographic characteristics between women who did not use hormonal contraceptives and women using hormonal contraceptives sub-categorised by route of administration (oral or non-oral formulation) and hormonal class (oestrogen/progestin combination or progestin-only).

## Suicidal behaviour

### First event of suicidal behaviour

Using Cox proportional hazard models, we calculated HRs and 95% CI of a first event of suicidal behaviour among women using distinct classes of hormonal contraceptives compared with women not using hormonal contraceptives. From this analysis, women with suicidal behaviour events before the start of follow-up were excluded. We used the calendar year as the underlying time scale, with adjustment for birth year and attained age, civil partnership, educational level, household income, parity and history of psychiatric disorder. We used robust standard errors accounting for the correlations between time periods within the same individual. Besides the relative measures of associations, we calculated

(IRDs) with 95% CIs adjusted for attained age.

### Risk modification by age

To estimate age-varying HRs of first event of suicidal behaviour, we applied restricted cubic splines to the attained age using four knots placed at the 0.05, 0.35, 0.65 and 0.95 quantiles of the distribution of outcome events and added interaction terms with the splined age.^29^ We further performed *post-hoc* stratified analyses by attained age (15–18 years, 19–29 years and 30–50 years).

### Repeated events of suicidal behaviour

To better control for unmeasured confounders, we conducted a within-individual analysis using fixed-term regression. Fixed-effects models rely on within-person variation, both on the exposure and outcome, to identify the effect of a time-varying exposure while limiting potential confounding by measured and unmeasured time-invariant factors and to account for correlations that arise between repeated measures.^29^ By design, this method uses the individual as her own control and compares the risk of suicidal behaviour between use and non-use periods, while adjusting for all factors that are constant within each individual during follow-up.^30 31^ Women with a history of suicide attempts were included, and repeated suicidal behaviour events were allowed in this analysis. Only women with both use and non-use periods, and at least one suicidal behaviour event, contributed to this analysis. Individuals who never used contraceptives, however, cannot contribute to analyses of exposure changes in contraceptive use. Moreover, individuals who started using contraceptives at any point during the study period were included in the analysis. The analysis was adjusted for time-varying covariates including attained age, parity, history of psychiatric disorder and history of suicidal attempt.

## Sensitivity analyses

We conducted three sensitivity analyses to examine whether the results of the first event of suicidal behaviour were altered by cohort selection and exposure definitions. The first analysis was restricted to a cohort of adolescents (born 1992–1998 and attained aged 15–18 years) and included only women who had no prior history of hormonal contraceptive use in order to assess the associations among individuals who were new to hormonal contraceptives. The second and third analyses were conducted to evaluate the potential influence of discontinuation, specifically regarding women who potentially discontinue due to mental discomfort, by using different methodological approaches. We examined the associations between hormonal contraceptives and suicidal behaviour by extending the end of each prescription by a 4-week period, so that any outcome following treatment discontinuation would also be considered under exposure period. For the third analysis, we estimated the risk of suicidal behaviour associated with *new use and prevalent use* of hormonal contraceptives, separately. We defined *new use* as any use of hormonal contraceptives that emerged after at least 1 year of no use, following the approach of the Danish study,^12^ while *prevalent use was defined* as using any type of contraceptive during the year prior. We considered *no use* (from both never users and former users) as the reference to independently compare with *new use* or *prevalent use*. The analysis was restricted from 1 January 2007 to allow for at least 1 year wash-out period for the definition of *new use*.

Several additional sensitivity analyses were conducted to assess the robustness of our findings. First, to examine whether psychiatric history moderates the association between hormonal contraceptive use and suicidal behaviour, we performed stratified analyses comparing women with and without a history of psychiatric disorders. Second, we conducted a focused analysis on suicide as a distinct outcome to allow for more precise interpretation. Third, we explored potential heterogeneity among non-oral progestin-only contraceptives.

## Depression diagnosis

We examined associations between hormonal contraceptive use and clinical diagnoses of depression using both the nationwide cohort, as described for the analysis of suicidal behaviour, and an independent Stockholm population-based cohort which we were able to complement with depression diagnoses from the Primary Care Register (Appendix 1). To assess potential risk modification by age, we performed stratified analyses by attained age (15–18 years, 19–29 years and 30–50 years).

Data preparation was performed using SAS version 9.4, SAS Institute Inc, Cary, NC, USA. Statistical analyses were performed using Stata version 15.1 (StataCorp LP, College Station, TX, USA). A p* *<0.05 was considered the threshold for statistical significance.

### Patient and public involvement

None.

## Results

In total, we included 2,039,988 women in the analysis. Of these, 68% had used at least one type of hormonal contraceptive (mean age 27.5±9.3 years), involving 12 393 561 person-years during the follow-up period (median 6.8 years). Women using non-oral progestin-only contraceptives had more children and psychiatric disorders compared with women using other types of hormonal contraceptives and non-users (table 1).

**Table 1:** Descriptive characteristics of women who never or ever used contraceptives

	No use	Oral		Non-oral	
		*Combined*	*Progestin-only*	*Combined*	*Progestin-only*
**During study period:**					
**Number of periods**	4 096 945	1 403 788	921 542	207 545	595 329
**Duration, mean**	2.0±2.4	1.3±1.4	1.0±1.2	0.7±0.8	2.3±1.9
**Person-years**	8 103 522	1 849 632	894 914	152 284	1 393 209
**At cohort entry:**					
**Number of women**	2 000 255	703 411	494 067	126 014	432 775
**Age, mean**	29.1±11.5	21.7±7.5	26.0±9.4	21.1±6.6	30.1±10.0
**Birth year**					
1956–1965	464 980 (23.2)	20 129 (2.9)	47 418 (9.6)	1957 (1.6)	80 357 (18.6)
1966–1975	472 062 (23.6)	87 524 (12.4)	118 455 (24.0)	12 747 (10.1)	155 478 (35.9)
1976–1985	417 691 (20.9)	213 163 (30.3)	153 416 (31.1)	41 704 (33.1)	92 394 (21.3)
1986–1998	645 522 (32.3)	382 595 (54.4)	174 778 (35.4)	69 606 (55.2)	104 546 (24.2)
**Civil partnership**					
No	1 253 631 (62.7)	497 093 (70.7)	343 138 (69.5)	91 905 (72.9)	253 322 (58.5)
Yes	486 858 (24.3)	58 350 (8.3)	86 815 (17.6)	9288 (7.4)	138 726 (32.1)
Unknown	259 766 (13.0)	147 968 (21.0)	64 114 (13.0)	24 821 (19.7)	40 727 (9.4)
**Education level**					
Low	263 726 (13.2)	90 803 (12.9)	49 527 (10.0)	12 785 (10.1)	45 224 (10.4)
Middle	875 922 (43.8)	308 431 (43.8)	229 807 (46.5)	55 314 (43.9)	213 186 (49.3)
High	807 278 (40.4)	296 813 (42.2)	211 643 (42.8)	57 427 (45.6)	170 045 (39.3)
Unknown	53 329 (2.7)	7364 (1.0)	3090 (0.6)	488 (0.4)	4320 (1.0)
**Individualised household income**					
Q1	496 877 (24.8)	181 935 (25.9)	131 731 (26.7)	31 983 (25.4)	121 493 (28.1)
Q2	501 053 (25.0)	192 245 (27.3)	130 755 (26.5)	34 363 (27.3)	118 489 (27.4)
Q3	491 137 (24.6)	190 764 (27.1)	126 790 (25.7)	35 951 (28.5)	102 033 (23.6)
Q4	470 195 (23.5)	133 671 (19.0)	103 495 (20.9)	23 551 (18.7)	89 891 (20.8)
Unknown	40 993 (2.0)	4796 (0.7)	1296 (0.3)	166 (0.1)	869 (0.2)
**Parity**					
0	1 133 548 (56.7)	594 705 (84.5)	316 945 (64.2)	106 706 (84.7)	174 961 (40.4)
1	207 159 (10.4)	40 415 (5.7)	59 731 (12.1)	7798 (6.2)	56 577 (13.1)
2	424 036 (21.2)	52 115 (7.4)	82 733 (16.7)	8799 (7.0)	137 411 (31.8)
3+	235 512 (11.8)	16 176 (2.3)	34 658 (7.0)	2711 (2.2)	63 826 (14.7)
**History of depression**					
No	1 864 469 (93.2)	673 792 (95.8)	465 680 (94.3)	119 941 (95.2)	400 699 (92.6)
Yes	135 786 (6.8)	29 619 (4.2)	28 387 (5.7)	6073 (4.8)	32 076 (7.4)
**History of any psychiatric disorder**					
No	1 777 397 (88.9)	646 174 (91.9)	445 571 (90.2)	114 217 (90.6)	381 213 (88.1)
Yes	222 858 (11.1)	57 237 (8.1)	48 496 (9.8)	11 797 (9.4)	51 562 (11.9)
**History of suicide behaviour**					
No	1 972 245 (98.6)	695 056 (98.8)	487 219 (98.6)	124 077 (98.5)	425 613 (98.3)
Yes	28 010 (1.4)	8355 (1.2)	6848 (1.4)	1937 (1.5)	7162 (1.7)

In total, we included 2 039 988 women. One woman could contribute to multiple groups.

## Suicidal behaviour

We identified 49 931 women with a first suicidal behaviour event during follow-up. The risk of suicidal behaviour was dependent on the class of hormonal contraceptives. Overall, no increased risk, but rather a protective association, was found between use of any oral contraceptives or non-oral combination formulations and a first suicidal behaviour event (eg oral combined contraceptives HR 0.82, 95% CI 0.79 to 0.84; table 2). In contrast, non-oral progestin-only contraceptive use was associated with an increased risk of a first suicidal behaviour event (HR 1.17, 95% CI 1.14 to 1.20). This corresponded to an age-adjusted incidence rate difference of 0.85 (95% CI 0.72 to 0.98) per 1000 person-years.

**Table 2:** Use of contraceptives and the risk of suicidal behaviour among all women, using first event in population analysis and repeated events with population and within-individual analyses

	*First event analysis*		
	N (IR)	Population-level HR (95% CI) *	IRD (95% CI) †
No use	33 434 (4.2)	1.0	0.00
Oral combined	6020 (3.3)	0.82 (0.79 to 0.84)	−1.26 (-1.37 to -1.15)
Oral progestin-only	3154 (3.6)	0.88 (0.85 to 0.92)	−0.69 (-0.83 to -0.56)
Non-oral combined	569 (3.8)	0.91 (0.84 to 99)	−0.73 (-1.05 to -0.40)
Non-oral progestin-only	6754 (5.0)	1.17 (1.14 to1.20)	0.85 (0.72 to 0.98)
	*Repeated events analysis*		
	N (IR)	Population-level HR (95% CI) *	Within-individual HR (95% CI) ‡
No use	80 610 (9.9)	1.0	1.0
Oral combined	10 381 (5.6)	0.80 (0.77 to 0.82)	1.05 (1.01 to 1.10)
Oral progestin-only	5945 (6.6)	0.89 (0.85 to 0.92)	1.04 (0.99 to 1.10)
Non-oral combined	955 (6.3)	0.82 (0.76 to 0.89)	1.00 (0.89 to 1.14)
Non-oral progestin-only	15 337 (11.0)	1.17 (1.13 to 1.21)	1.16 (1.11 to 1.21)

* The estimates were adjusted for year of birth, attained age, civil partnership, educational level, household income, parity, history of psychiatric disorder and additionally history of suicidal behaviour in analysis of repeated events.

† IRDs were adjusted for attained age (15–18 and every 5 years thereafter).

‡ The estimates were adjusted for attained age, parity, history of psychiatric disorder and history of suicidal behaviour.

IR, crude incidence rate (per 1000 person-years); IRD, incidence rate differences.

In addition to the first event analysis, we also examined associations between hormonal contraceptive use and repeated suicidal events. In the population-based analysis, the results for repeated suicidal events were comparable to first suicidal event outcomes. Using a within-individual design, the protective association observed in the population-based analysis between use of oral contraceptives or non-oral combination formulations with suicidal attempts was no longer present. In particular, use of oral combined contraceptives was associated with a slightly elevated risk of suicidal attempts in the within-individual analysis (HR 1.05, 95% CI 1.01 to 1.10; table 2). The associated risk of non-oral progestin-only contraceptive use with suicidal behaviour observed in the population-based analysis was replicated in the within-individual analysis (HR 1.16, 95% CI 1.11 to 1.21).

### Risk modification by age

**Figure 1:**
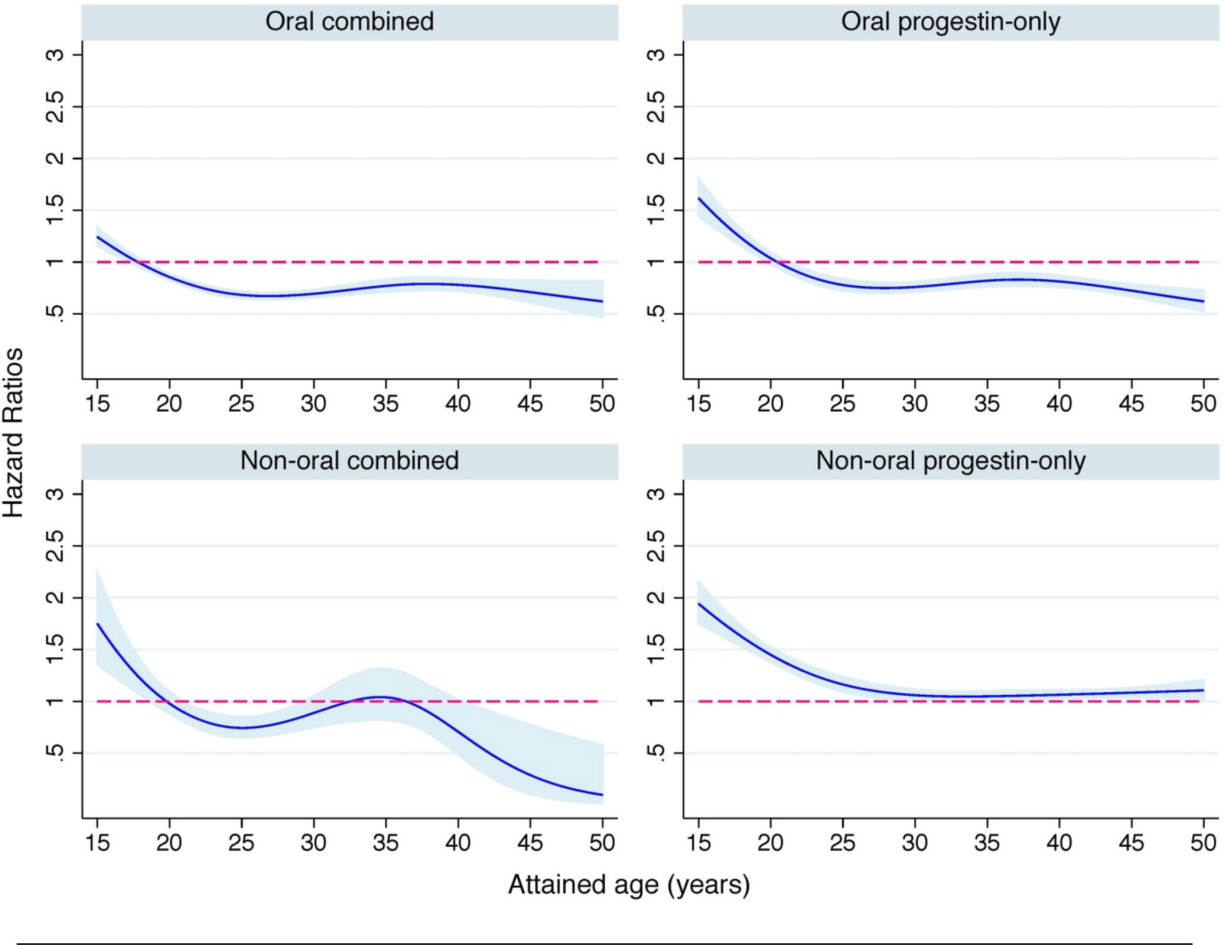
Use of contraceptives and the subsequent risk of suicidal behaviour across attained age using first event in population analysis. The estimates were adjusted for year of birth, attained age, civil partnership, educational level, household income, parity and history of psychiatric disorder.

An age-dependent association was observed between hormonal contraception and first suicidal behaviour event (figure 1). During late adolescence (15–18 years), use of any class of hormonal contraception was associated with a higher risk of suicidal behaviour (range of HRs: 1.09–1.80; table 3). In adult ages, the associations attenuated and became protective for oral contraceptives and non-oral combined formulations (figure 1, table 3). However, the risk of suicidal behaviour remained elevated in women using non-oral progestin-only contraception throughout the entire window of reproductive ages. Risk estimates were higher in younger (HR 1.80, 95% CI 1.67 to 1.94) vs older (HR 1.07, 95% CI 1.03 to 1.10) women (table 3).

**Table 3:** Use of contraceptives and the risk of suicidal behaviour among all women, stratified by attained age, using first event in population analysis

	*Population analysis*		
	N (IR)	HR (95% CI)	IRD (95% CI)
**Age 15–18** years			
No use	5463 (4.6)	1.0	0.00
Oral combined	2207 (4.9)	1.09 (1.03 to 1.15)	0.28 (0.04 to 0.52)
Oral progestin-only	682 (6.8)	1.36 (1.26 to 1.48)	2.16 (1.63 to 2.68)
Non-oral combined	134 (6.9)	1.35 (1.13 to 1.60)	2.25 (1.08 to 3.42)
Non-oral progestin-only	836 (11.4)	1.80 (1.67 to 1.94)	6.75 (5.97 to 7.53)
**Age 19–29** years			
No use	8683 (4.7)	1.0	0.00
Oral combined	2886 (2.9)	0.74 (0.71 to 0.78)	−1.96 (-2.11 to -1.81)
Oral progestin-only	1156 (3.7)	0.86 (0.81 to 0.91)	−1.06 (-1.29 to -0.82)
Non-oral combined	332 (3.4)	0.81 (0.73 to 0.91)	−1.41 (-1.79 to -1.03)
Non-oral progestin-only	1611 (6.6)	1.22 (1.15 to 1.28)	1.80 (1.46 to 2.13)
**Age 30–50** years			
No use	19 288 (4.0)	1.0	0.00
Oral combined	927 (2.5)	0.74 (0.69 to 0.79)	−1.27 (-1.45 to -1.09)
Oral progestin-only	1316 (2.9)	0.77 (0.73 to 0.82)	−1.07 (-1.23 to -0.90)
Non-oral combined	103 (3.3)	0.94 (0.77 to 1.14)	−0.46 (-1.11 to 0.18)
Non-oral progestin-only	4307 (4.2)	1.07 (1.03 to 1.10)	0.20 (0.06 to 0.34)

HRs were adjusted for year of birth, attained age, civil partnership, educational level, household income, parity and history of psychiatric disorder.

IRDs were adjusted for attained age (15–18 and every 5 year thereafter).

IR, crude incidence rate (per 1000 person-years); IRD, Incidence Rate Differences (per 1000 person-years).

## Sensitivity analyses

Evaluation of the subsample of women aged 15–18 years with no history of contraceptive use before cohort entry yielded similar findings as the main analysis in adolescents (online supplemental table S2). A sensitivity analysis for the association between suicidal behaviour and hormonal contraceptive use, in which prescription periods for every treatment were extended by 4 weeks, was comparable to the main analyses (online supplemental table S3). Likewise, redefining exposure periods as new-users vs prevalent-users also yielded similar results as the main population-based analysis regarding associations between suicidal behaviour and hormonal contraceptive use, including when stratified by attained age (online supplemental table S4).

Additional sensitivity analyses showed that the link between hormonal contraception and suicidal behaviour was consistent regardless of psychiatric history (online supplemental table S5). Suicide-specific analyses were limited by sample size but did not reveal any association between hormonal contraceptive use and death by suicide (online supplemental table S6). Finally, among distinct formulations of non-oral progestin-only contraceptives, we observed an elevated risk of a first event of suicidal behaviour in association with the use of gonane intrauterine device (IUDs) and implants, whereas pregnane-based progestin injections were not associated with increased risk (online supplemental table S7).

## Depression diagnosis

Overall, no increased risk of depression diagnosis was observed with distinct classes of hormonal contraceptive use in either the nationwide or Stockholm cohorts (table 4). Rather, both classes of oral contraceptives demonstrated a significantly protective association with depression diagnosis (Nationwide cohort – oral combined: HR 0.78, 95% CI 0.76 to 0.80; oral progestin-only: HR 0.75, 95% CI 0.71 to 0.78).

**Table 4:** Use of contraceptives and the risk of depression diagnosis among all women, stratified by attained age, in the nationwide and Stockholm population-based cohorts

		Nationwide cohort		Stockholm cohort	
		N (IR)	HR (95% CI)	N (IR)	HR (95% CI)
**Overall**					
No use		30 189 (4.2)	1.00	19 316 (12.3)	1.00
Oral combined pill		6410 (3.8)	0.78 (0.76 to 0.80)	3444 (10.4)	0.85 (0.82 to 0.89)
Oral progestin-only pill		2382 (3.0)	0.75 (0.71 to 0.78)	1417 (9.5)	0.78 (0.74 to 0.83)
Non-oral combined		649 (4.8)	0.99 (0.92 to 1.07)	527 (15.0)	1.09 (1.00 to 1.19)
Non-oral progestin-only		4049 (3.4)	0.94 (0.90 to 0.97)	2245 (12.2)	0.98 (0.94 to 1.03)
**Age 15–18** years					
No use		7386 (6.3)	1.0	1958 (8.4)	1.0
Oral combined pill		2722 (6.2)	1.04 (0.99 to 1.09)	691 (10.3)	1.18 (1.08 to 1.30)
Oral progestin-only pill		792 (8.1)	1.23 (1.14 to 1.33)	161 (10.1)	1.14 (0.97 to 1.34)
Non-oral combined		181 (9.6)	1.40 (1.20 to 1.62)	77 (15.9)	1.43 (1.13 to 1.80)
Non-oral progestin-only		948 (13.4)	1.54 (1.44 to 1.65)	194 (19.9)	1.63 (1.40 to 1.89)
**Age 19–29** years					
No use		9625 (5.6)	1.0	6050 (15.4)	1.0
Oral combined pill		3035 (3.2)	0.69 (0.66 to 0.72)	2064 (10.6)	0.79 (0.75 to 0.83)
Oral progestin-only pill		993 (3.4)	0.69 (0.65 to 0.74)	652 (11.2)	0.78 (0.72 to 0.85)
Non-oral combined		399 (4.5)	0.92 (0.83 to 1.01)	370 (15.7)	1.06 (0.95 to 1.17)
Non-oral progestin-only		1364 (6.2)	1.00 (0.95 to 1.06)	652 (17.3)	1.01 (0.93 to 1.10)
**Age 30–50** years					
No use		13 178 (3.1)	1.0	11 308 (11.9)	1.0
Oral combined pill		653 (2.0)	0.69 (0.63 to 0.74)	689 (10.2)	0.86 (0.79 to 0.93)
Oral progestin-only pill		597 (1.5)	0.54 (0.50 to 0.59)	604 (8.1)	0.71 (0.66 to 0.78)
Non-oral combined		69 (2.6)	0.89 (0.70 to 1.13)	80 (12.0)	0.97 (0.78 to 1.21)
Non-oral progestin-only		1737 (1.9)	0.72 (0.68 to 0.76)	1399 (10.2)	0.91 (0.86 to 0.96)

The estimates were adjusted for year of birth, attained age, civil partnership, educational level, household income, parity, and history of psychiatric disorder (other than depression).

IR, crude incidence rate (per 1000 person-years).;

We observed a risk modification by age (table 4, online supplemental figure S1). Oral progestin-only, non-oral progestin-only and non-oral combined formulations were each associated with an elevated risk of depression diagnosis during late adolescence (15–18 years; range of HRs: 1.23–1.54). This increased risk did not persist into adulthood for any of the hormonal contraceptive formulations (19–29 years old, 30–50 years old). Moreover, oral combination, oral progestin-only and non-oral progestin-only each exhibited a protective association with depression diagnosis in adulthood (30–50 years; range of HRs: 0.54–0.72).

## Discussion

In a nationwide cohort study involving over two million women of reproductive age, we found no consistent association between oral hormonal contraception or non-oral combination formulations with risk of suicidal behaviour. In contrast, non-oral progestin-only contraception was associated with an elevated risk of suicidal behaviour. No increased risk of depression diagnosis was observed for any class of hormonal contraception. Age-stratified analyses revealed a higher risk of suicidal behaviour and depression in association with use of any class of hormonal contraceptive during adolescence, most of which declined to null or protective in adulthood.

Overall, when including women across the entire range of reproductive ages, our results suggest no association between the use of oral hormonal contraceptives on the risk of depression and suicidal behaviour – findings that do not replicate the previously reported associations between hormonal contraception and adverse mental health. In adolescents, however, the elevated risk of suicidal behaviour and depression across all forms of hormonal contraceptives is consistent with the results reported in the Danish studies.^15 16^

It is plausible that hormonal contraceptives exert a physiological influence on the risk of suicidal behaviour, possibly reflecting an enhanced vulnerability to exogenous hormone exposure in adolescents. Considering that gonadal hormones exert a powerful influence on brain development and maturation,^31^ exposure to hormonal contraceptives may inadvertently result in adverse mental health outcomes.^32^ While undergoing physiological changes, adolescents and young adults are also addressing new psychosocial challenges, such as developing self-esteem or building intimate relationships. Hence, given that use of hormonal contraceptives is a proxy for sexual activity,^20^ and younger age of initiation of sexual activity has been associated with elevated rates of risk-taking behaviour, self-harm and adverse health outcomes,^33^ the association of hormonal contraceptive use with suicidal behaviour may exhibit confounding by indication. Furthermore, specific indications for contraceptive prescriptions in adolescents, such as dysmenorrhoea, polycystic ovary syndrome and endometriosis, are associated with a higher risk of depression and suicidality.

While medical risks and side effects of oestrogen influence some women to choose progestin-only contraceptives, the widespread use of these methods—particularly implants and IUDs—is largely driven by their effectiveness and convenience as long-acting reversible contraceptives (LARCs). Our analysis showed a consistent pattern of non-oral progestin-only contraception being more strongly associated with suicidal behaviour in both adolescents and older women. Most notably, these formulations, except for pregnane-based progestins, were associated with suicidal behaviour across the entire reproductive lifespan. Non-oral progestin-only contraceptives have previously been demonstrated to potentiate the systemic physiological responses to stress, including excessive heart rate responsivity and exaggerated cortisol responses, a finding not observed for oestrogen/progestin combination formulations, and thereby suggesting a candidate neurobiological mechanism underlying the observed associations with suicidality.^34 35^

Hormonal contraceptives, in particular non-oral progestin-only formulations, are frequently prescribed for treatment of somatic disorders or symptoms including dysmenorrhoea, severe menstrual pain, acne, premenstrual syndrome or dysphoric disorder and endometriosis – conditions that might adversely influence mood and risk of self-harm, and therefore could be an instance of confounding by indication.^22 36 37^ Moreover, risk-taking behaviour, self-harm and mood disorders are also influenced by genetic and early developmental factors.^38^ Furthermore, the limited availability of covariates, especially for co-morbid conditions including migraine with aura and epilepsy, may have also influenced the findings. However, this is for the within-individual comparison, unless it is a new condition that emerged between two follow-up periods. The within-individual comparison was designed to attenuate such unmeasured potential confounders, assuming they are stable across time. Furthermore, the different risk profiles of distinct contraceptive methods could also be influenced by confounding by indication, for example, among women with thromboembolic diseases with a contraindication for use of oestrogen/progestin combination formulations, or in women with prescription adherence challenges due to specific underlying health conditions.^4^ Relationship status and family planning may also influence both use and choice of contraceptives,^39^ as well as mental health.^40^ Although we adjusted for rates of civil partnership, which included marriage and registered partnerships, we cannot rule out residual confounding by unregistered romantic relationships. Further research is needed to translate these and related findings into an evidence-based approach to personalised contraception guided by a thorough understanding of the underlying biology, clinically reliable biomarkers and careful consideration of ethical issues.

The observed pattern of declining risk with increasing age for suicidal behaviour and depression among women using hormonal contraceptives may partly be explained by the use of non-exposed periods (both never-users and former-users), rather than only never-users, as a reference. Our decision to utilise non-exposed periods as the reference condition was predicated on the overall aim of the study to investigate the association between the use of hormonal contraception and subsequent suicidal behaviour across the entire window of reproductive ages, given that hormonal contraception is widely utilised for family planning by women throughout all reproductive ages. Consequently, women who have never used hormonal contraception may also be a highly selected group.

Moreover, the finding of declining risk with increasing age may be related to selective discontinuation, ie, a tendency of women to discontinue treatment or switch to other methods if they experience adverse effects of hormonal contraceptives,^40^ while those without symptoms preferentially continue use into adulthood. In contrast to oral hormonal contraceptives, however, discontinuation of non-oral progestin-only contraceptives, including intrauterine devices and implants, typically requires the assistance of a healthcare provider, resulting in a higher barrier for termination compared with other reversible contraceptive methods.^41^ Furthermore, we observed consistent associations across multiple sensitivity analyses including the incident user subcohort, extended treatment period definition and comparison between new users and prevalent users. All those additional analyses together provide additional support for our main findings regarding the declining risk of suicidal behaviour among middle-aged women using hormonal contraception.

The strengths of this study included a nationwide sample of more than two million women, longitudinal cohort design and objective measures of exposures and outcomes. The sample size provided sufficient statistical power to explore associations between distinct hormonal contraceptive formulations and suicidal behaviour using a within-individual comparison design, which has been shown to offer a powerful approach to control for sustained unmeasured confounders,^42^ such as gynaecological conditions and subclinical mental health problems.

Nevertheless, the present findings should be considered in light of some limitations. First, reliance on prescription data for hormonal contraceptive use has been well-validated,^43^ but is still a proxy metric which has a temporal resolution on the order of months. Second, information regarding less severe suicidal behaviour that did not require medical care was incomplete. But such misclassification should be non-differential across exposures and would be expected to have attenuated the associations. Third, the limited availability of comprehensive primary care data presents a significant limitation, which may have obscured the full spectrum of mental health conditions and treatments, including those related to suicidal behaviour, and impacted estimates of the reported associations. Fourth, we acknowledge that the available years of data from the prescribed drug register are limited, for which it was not possible to determine whether older women are new users, thereby restricting the possibility of properly addressing the healthy bias issue in women of older reproductive age. Yet in the context of high rates of suicides among middle-aged women, prevalent use of hormonal contraceptives among women of older reproductive age is a relevant public health topic that should continue to be addressed in future studies. Additionally, while we categorised contraceptives based on their hormonal composition and administration routes, considerable variability exists within these categories, potentially influencing the interpretation of their association with mental health outcomes. Although the study was not designed or powered to assess each specific subclass of non-oral progestin-only contraceptives, our preliminary analyses indicated an association between gonane-containing IUDs and suicidal behaviour (online supplemental table S7), suggesting that gonane-containing implants may carry an even higher risk than other non-oral formulations. In contrast, pregnane-based injections were not associated with an increased risk. A complementary sensitivity analysis that focused specifically on death by suicide (online supplemental table S6) did not show a significant association with hormonal contraceptive use overall. These findings suggest that while certain formulations may be linked to suicidal behaviour, including self-harm, the relationship with death by suicide remains uncertain and warrants further investigation in larger, more adequately powered studies. Future research should not only explore the physiological mechanisms of contraceptive subclasses but also consider differences across specific mental health conditions, given that their associations with hormonal contraceptive use and suicidality are unlikely to be uniform. Such studies could provide a more comprehensive perspective on mental health outcomes and yield nuanced insights to inform clinical decision-making. We also acknowledge that our findings may not fully reflect evolving demographic trends of hormonal contraceptive use and their potential relationship with suicidal behaviour. Future research focusing on recently approved formulations, including lower-dose IUDs, will be crucial to further inform clinical practice and evidence-based guidelines. Finally, we note that combining different types of suicidal behaviours into a single category may have limited the ability to fully understand the nuanced relationship between hormonal contraceptive use and suicide risk.

This nationwide longitudinal study found no overall increased risk of suicidal behaviour among women using oral contraceptives or non-oral combined formulations. Risk modification by age demonstrated novel clinically important associations in older women, as well as replicating previous risk estimates in young women. The elevated risk of suicidal behaviour associated with hormonal contraceptives during adolescence and non-oral progestin-only contraception—particularly gonane-containing formulations— across the entire range of reproductive ages warrants attention and further investigation. Women should be counselled by their healthcare clinicians regarding the associated risks of suicidal behaviour among distinct hormonal formulations as part of patient-centred discussions surrounding contraceptive choice.

## Supplementary material

10.1136/bmjopen-2025-105694 online supplemental file 1

10.1136/bmjopen-2025-105694 online supplemental figure 1

## Data Availability

Access to the data may be granted upon reasonable request to the Swedish National Board of Health and Welfare, subject to applicable legal and ethical regulations.

## References

[1] United Nations (2015). Department of economic and social affairs. 2015: trends in contraceptive use worldwide.

[2] Reid R, Leyland N, Wolfman W (2010). SOGC clinical practice guidelines: Oral contraceptives and the risk of venous thromboembolism: an update. Int J Gynaecol Obstetrics.

[3] Plu-Bureau G, Maitrot-Mantelet L, Hugon-Rodin J (2013). Hormonal contraceptives and venous thromboembolism: an epidemiological update. Best Pract Res Clin Endocrinol Metab.

[4] Gaffield ML, Kiarie J (2016). WHO medical eligibility criteria update. Contraception.

[5] Toffol E, Heikinheimo O, Koponen P (2011). Hormonal contraception and mental health: results of a population-based study. Hum Reprod.

[6] Worly BL, Gur TL, Schaffir J (2018). The relationship between progestin hormonal contraception and depression: a systematic review. Contraception.

[7] Vessey MP, Villard-Mackintosh L, McPherson K (1989). Mortality among oral contraceptive users: 20 year follow up of women in a cohort study. BMJ.

[8] Beral V, Hermon C, Kay C (1999). Mortality associated with oral contraceptive use: 25 year follow up of cohort of 46 000 women from Royal College of General Practitioners’ oral contraception study. BMJ.

[9] Colditz GA (1994). Oral contraceptive use and mortality during 12 years of follow-up: the Nurses’ Health Study. Ann Intern Med.

[10] Hannaford PC, Iversen L, Macfarlane TV (2010). Mortality among contraceptive pill users: cohort evidence from Royal College of General Practitioners’ Oral Contraception Study. BMJ.

[11] Charlton BM, Rich-Edwards JW, Colditz GA (2014). Oral contraceptive use and mortality after 36 years of follow-up in the Nurses’ Health Study: prospective cohort study. BMJ.

[12] Skovlund CW, Mørch LS, Kessing LV (2016). Association of Hormonal Contraception With Depression. JAMA Psychiatry.

[13] Skovlund CW, Mørch LS, Kessing LV (2018). Association of Hormonal Contraception With Suicide Attempts and Suicides. Am J Psychiatry.

[14] Jung SJ, Cho SMJ, Kim HC (2019). Association of oral contraceptive use with suicidal behavior among representative Korean population: Results from Korea National Health and Nutrition Examination Survey (2007-2016). J Affect Disord.

[15] Pérez-López FR, Pérez-Roncero GR, López-Baena MT (2020). Hormonal contraceptives and the risk of suicide: a systematic review and meta-analysis. European Journal of Obstetrics & Gynecology and Reproductive Biology.

[16] Edwards AC, Lönn SL, Crump C (2022). Oral contraceptive use and risk of suicidal behavior among young women. Psychol Med.

[17] Toffol E, Partonen T, Heikinheimo O (2024). Use of systemic hormonal contraception and risk of attempted suicide: a nested case-control study. Eur J Epidemiol.

[18] Booij HS, Giltay EJ, Joffe H (2019). Association of Use of Oral Contraceptives With Depressive Symptoms Among Adolescents and Young Women. JAMA Psychiatry.

[19] Parellada M, Saiz P, Moreno D (2008). Is attempted suicide different in adolescent and adults?. Psychiatry Res.

[20] United Nations Department of Economic and Social Affairs, Population Division World family planning 2020 highlights: accelerating action to ensure universal access to family planning (st/esa/ser.a/450).

[21] National Institute of Mental Health (2020) (2020). Suicide. https://www.nimh.nih.gov/health/statistics/suicide.shtml.

[22] Elovainio M, Teperi J, Aalto A-M (2007). Depressive symptoms as predictors of discontinuation of treatment of menorrhagia by levonorgestrel-releasing intrauterine system. Int J Behav Med.

[23] Ludvigsson JF, Otterblad-Olausson P, Pettersson BU (2009). The Swedish personal identity number: possibilities and pitfalls in healthcare and medical research. Eur J Epidemiol.

[24] Wettermark B, Hammar N, Fored CM (2007). The new Swedish Prescribed Drug Register--opportunities for pharmacoepidemiological research and experience from the first six months. Pharmacoepidemiol Drug Saf.

[25] Tidemalm D, Långström N, Lichtenstein P (2008). Risk of suicide after suicide attempt according to coexisting psychiatric disorder: Swedish cohort study with long term follow-up. BMJ.

[26] Hirvikoski T, Boman M, Chen Q (2020). Individual risk and familial liability for suicide attempt and suicide in autism: a population-based study. Psychol Med.

[27] Harrell F (2015). Regression modeling strategies: with applications to linear models, logistic regression, and survival analysis. J Am Stat Assoc Second Edi Springer Series in Statistics.

[28] Simon GE, Johnson E, Lawrence JM (2018). Predicting Suicide Attempts and Suicide Deaths Following Outpatient Visits Using Electronic Health Records. Am J Psychiatry.

[29] Keen R, Chen JT, Slopen N (2023). Prospective Associations of Childhood Housing Insecurity With Anxiety and Depression Symptoms During Childhood and Adulthood. JAMA Pediatr.

[30] Lindberg M, Foldemo A, Josefsson A (2012). Differences in prescription rates and odds ratios of antidepressant drugs in relation to individual hormonal contraceptives: A nationwide population-based study with age-specific analyses. Eur J Contracept Reprod Health Care.

[31] Dahl RE, Allen NB, Wilbrecht L (2018). Importance of investing in adolescence from a developmental science perspective. Nature New Biol.

[32] van Heeringen K, Mann JJ (2014). The neurobiology of suicide. Lancet Psychiatry.

[33] Aleknaviciute J, Tulen JHM, De Rijke YB (2017). The levonorgestrel-releasing intrauterine device potentiates stress reactivity. Psychoneuroendocrinology.

[34] Lara LAS, Abdo CHN (2016). Age at Time of Initial Sexual Intercourse and Health of Adolescent Girls. J Pediatr Adolesc Gynecol.

[35] Laganà AS, La Rosa VL, Rapisarda AMC (2017). Anxiety and depression in patients with endometriosis: impact and management challenges. Int J Womens Health.

[36] Ferro MA, Rhodes AE, Kimber M (2017). Suicidal Behaviour Among Adolescents and Young Adults with Self-Reported Chronic Illness. Can J Psychiatry.

[37] Althoff RR, Hudziak JJ, Willemsen G (2017). Genetic and environmental contributions to self‐reported thoughts of self‐harm and suicide. American J of Med Genetics Pt B.

[38] Harvey SM, Oakley LP, Washburn I (2018). Contraceptive Method Choice Among Young Adults: Influence of Individual and Relationship Factors. J Sex Res.

[39] Braithwaite S, Holt-Lunstad J (2017). Romantic relationships and mental health. Curr Opin Psychol.

[40] Amico JR, Bennett AH, Karasz A (2017). “I wish they could hold on a little longer”: physicians’ experiences with requests for early IUD removal. Contraception.

[41] Johnson S, Pion C, Jennings V (2013). Current methods and attitudes of women towards contraception in Europe and America. Reprod Health.

[42] Lindh I, Skjeldestad FE, Gemzell-Danielsson K (2017). Contraceptive use in the Nordic countries. Acta Obstet Gynecol Scand.

[43] Lichtenstein P, Halldner L, Zetterqvist J (2012). Medication for attention deficit-hyperactivity disorder and criminality. N Engl J Med.

