# Supplementary material for: Hormonal contraception and the risk of suicidal behaviour: a Swedish nationwide register-based study
Source: BMJ Open. 2025 Nov 27;15(11):e105694. doi: 10.1136/bmjopen-2025-105694 (PMC12666169; doi:10.1136/bmjopen-2025-105694)
Supplement: online supplemental file 1 [file bmjopen-15-11-s001.docx]

**Table S1. Hormonal contraception, corresponding codes and daily defined dose**

| Hormonal contraception groups | | | ATC codes | DDD |
| --- | --- | --- | --- | --- |
| **Progestin**  **Class** | **Active Ingredient** | **Formulation** |  |  |
| **Oral route of administration** | | |  |  |
| *Combined products* (Ethinylestradiol + Progestins) | | | |  |
| Estrane | Norethisterone, Lynestrenol, Dienogest | Tablet | G03AA03, G03AB02, G03AA05, G03AB04, G03AB08 | 84/168/364 |
| Gonane | Levonorgestrel, Desogestrel, Norgestimate |  | G03AA07, G03AB03, G03AA09, G03AB05, G03AA11 | 84/168/364 |
| Pregnane | Nomegestrol |  | G03AA14 | 84 |
| Non-steroidal | Drospirenone |  | G03AA12 | 84 |
| *Progestin-only products* | | |  |  |
| Estrane | Norethisterone, Lynestrenol | Tablet | G03AC01, G03AC02 | 84 |
| Gonane | Levonorgestrel, Desogestrel |  | G03AC03, G03AC09 | 84/168 |
|  | | |  |  |
| **Non-oral route of administration** | | |  |  |
| *Combined products* (Ethinylestradiol + Progestins) | | |  |  |
| Gonane | Etonogestrel | Vaginal ring | G02BB | 1 or 3 mos |
| Gonane | Norelgestromin | Transdermal patch | G03AA13 | 1 or 3 mos |
| *Progestin-only products* | | | | |
| Gonane | Levonorgestrel | IUD | G02BA03 | 5/3 yrs |
| Gonane | Etonogestrel | Implant | G03AC08 | 3yrs |
| Pregnane | Medroxyprogesterone | Injection | G03AC06 | 3 mos |

DDD, Daily Defined Dose; ATC, Anatomical Therapeutic Chemical classification system; IUD; Intrauterine device.

**Table S2. Sensitivity analysis for use of contraceptives and the risk of first suicidal behavior among women born during 1992-1998 and with attained age of 15-18 years. #**

|  | *Population analysis* | |
| --- | --- | --- |
|  | N(IR) | HR (95% CI) ^*^ |
| No use | 2,945 (4.2) | 1.0 |
| Oral combined | 944 (4.9) | 1.20 (1.10-1.29) |
| Oral progestin-only | 302 (6.7) | 1.39 (1.23-1.57) |
| Non-oral combined | 62 (6.4) | 1.28 (0.99-1.66) |
| Non-oral progestin-only | 328 (10.5) | 1.57 (1.39-1.77) |

N, number of events; IR, crude incidence rate (per 1,000 person-years); HR, hazard ratio; CI, confidence interval.

* The estimates were adjusted for year of birth, attained age, educational level, household income and history of psychiatric disorder.

# In this analysis, we restricted to individuals with no use of contraceptives at the cohort entry but may initiate a prescription later during the follow-up (i.e., incident users).

**Table S3. Sensitivity analysis for use of contraceptives and the risk of suicidal behavior among all women by extending contraceptive prescription by four weeks, using first event in population analysis.**

|  | *Population analysis* | |
| --- | --- | --- |
|  | N(IR) | HR (95% CI) ^*^ |
| No use | 32,522 (4.2) | 1.0 |
| Oral combined | 6,436 (3.4) | 0.84 (0.82-0.87) |
| Oral progestin-only | 3,447 (3.7) | 0.92 (0.88-0.95) |
| Non-oral combined | 653 (4.1) | 0.97 (0.90-1.05) |
| Non-oral progestin-only | 6,873 (5.0) | 1.18 (1.15-1.21) |

N, number of events; IR, crude incidence rate (per 1,000 person-years); HR, hazard ratio; CI, confidence interval.

^*^ The estimates were adjusted for year of birth, attained age, civil partnership, educational level, household income, parity, history of psychiatric disorder, and additionally history of suicidal behavior in analysis of repeated events.

^#^ The estimates were adjusted for attained age, parity, history of psychiatric disorder, and history of suicidal behavior.

**Table S4. New uses and prevalent uses of contraceptives and the risk of suicidal behavior among all women, stratified by attained age, using first event in population analysis.**

|  |  | | New use | | | | Prevalent use | | | |
| --- | --- | --- | --- | --- | --- | --- | --- | --- | --- | --- |
|  |  | | N (IR) | | HR* (95% CI) | | N (IR) | | HR* (95% CI) | |
| **Overall** | |  | |  | |  | |  | |  |
| No use | | *29,247 (4.3)* | | *1.0* | | *29,247 (4.3)* | | *1.0* | |  |
| Oral combined pill | | 2,569 (3.1) | | 0.76 (0.73-0.80) | | 2,732 (3.7) | | 0.87 (0.83-0.90) | |  |
| Oral progestin-only pill | | 1,172 (3.4) | | 0.87 (0.82-0.93) | | 1,716 (3.8) | | 0.89 (0.85-0.94) | |  |
| Non-oral combined | | 132 (3.7) | | 0.88 (0.74-1.05) | | 390 (3.9) | | 0.90 (0.81-1.00) | |  |
| Non-oral progestin-only | | 4,163 (4.7) | | 1.16 (1.12-1.20) | | 2,234 (5.9) | | 1.21 (1.16-1.26) | |  |
| **Age 15-18 yrs** | | |  | |  | |  | |  | |
| No use | | | *4,691 (4.6)* | | *1.0* | | *4,691 (4.6)* | | *1.0* | |
| Oral combined pill | | | 1,199 (4.5) | | 1.03 (0.96-1.10) | | 749 (5.9) | | 1.25 (1.15-1.35) | |
| Oral progestin-only pill | | | 312 (6.5) | | 1.35 (1.20-1.52) | | 318 (7.4) | | 1.45 (1.30-1.63) | |
| Non-oral combined | | | 31 (6.5) | | 1.33 (0.93-1.89) | | 91 (6.8) | | 1.34 (1.09-1.65) | |
| Non-oral progestin-only | | | 314 (12.6) | | 1.88 (1.67-2.11) | | 461 (10.6) | | 1.79 (1.62-1.97) | |
| **Age 19-29 yrs** | | |  | |  | |  | |  | |
| No use | | | *7,648 (4.7)* | | *1.0* | | *7,648 (4.7)* | | *1.0* | |
| Oral combined pill | | | 1,030 (2.5) | | 0.67 (0.63-0.71) | | 1,507 (3.3) | | 0.80 (0.76-0.85) | |
| Oral progestin-only pill | | | 377 (3.6) | | 0.84 (0.76-0.93) | | 675 (3.8) | | 0.86 (0.79-0.93) | |
| Non-oral combined | | | 72 (3.4) | | 0.80 (0.63-1.01) | | 233 (3.3) | | 0.80 (0.70-0.91) | |
| Non-oral progestin-only | | | 758 (6.9) | | 1.26 (1.17-1.36) | | 769 (6.4) | | 1.20 (1.11-1.29) | |
| **Age 30-50 yrs** | | |  | |  | |  | |  | |
| No use | | | *16,908 (4.1)* | | *1.0* | | *16,908 (4.1)* | | *1.0* | |
| Oral combined pill | | | 340 (2.2) | | 0.67 (0.60-0.75) | | 476 (3.0) | | 0.80 (0.73-0.87) | |
| Oral progestin-only pill | | | 483 (2.6) | | 0.74 (0.68-0.81) | | 723 (3.2) | | 0.79 (0.73-0.85) | |
| Non-oral combined | | | 29 (2.9) | | 0.84 (0.58-1.21) | | 66 (3.6) | | 0.99 (0.78-1.26) | |
| Non-oral progestin-only | | | 3,091 (4.1) | | 1.07 (1.03-1.11) | | 1,004 (4.7) | | 1.07 (1.00-1.14) | |

N, number of events; IR, crude incidence rate (per 1,000 person-years); HR, hazard ratio; CI, confidence interval.

^*^ The estimates were adjusted for year of birth, attained age, civil partnership, educational level, household income, parity, history of psychiatric disorder, and additionally history of suicidal behavior in analysis of repeated events.

| **Table S5. Use of contraceptives and the subsequent risk of suicide behavior in the nationwide population-based cohort, using first event in population analysis.** HRs in population analysis were derived from Cox proportional hazards model using calendar year as underlying time scale, and adjusted for birth year (every 10 years during 1956-1985, and 1986-1998), attained age, marriage status (married, unmarried, or unknown), education level (primary school, high school, college+, or unknown), individualized household income (four quartiles or unknown), parity (0, 1, 2, or 3+), and history of mental disorder (yes or no),, and accounted for within-individual correlations using robust variance estimates. | | |
| --- | --- | --- |
|  | N (IR) | HR (95% CI) |
| **All women** |  |  |
| No use | 33,413 (4.2) | 1.0 |
| Oral combined | 6,021 (3.3) | 0.82 (0.79-0.84) |
| Non-oral combined | 567 (3.8) | 0.91 (0.83-0.99) |
| Oral progestin alone | 3,140 (3.6) | 0.88 (0.85-0.92) |
| Non-oral progestin alone |  |  |
| LNG-IUD | 4,097 (4.4) | 1.15 (1.11-1.19) |
| Implant/Injection | 2,693 (6.5) | 1.20 (1.16-1.25) |
| **Without history of depression** |  |  |
| No use | 18,640 (2.8) | 1.0 |
| Oral combined | 3,927 (2.4) | 0.86 (0.83-0.90) |
| Non-oral combined | 335 (2.6) | 0.96 (0.86-1.08) |
| Oral progestin alone | 1,976 (2.6) | 0.95 (0.91-1.00) |
| Non-oral progestin alone |  |  |
| LNG-IUD | 2,373 (3.1) | 1.18 (1.13-1.24) |
| Implant/Injection | 1,478 (4.3) | 1.30 (1.24-1.38) |
| **With history of depression** |  |  |
| No use | 14,773 (12.2) | 1.0 |
| Oral combined | 2,094 (13.0) | 0.77 (0.74-0.81) |
| Non-oral combined | 232 (12.3) | 0.79 (0.70-0.91) |
| Oral progestin alone | 1,164 (10.2) | 0.78 (0.73-0.83) |
| Non-oral progestin alone |  |  |
| LNG-IUD | 1,724 (10.3) | 1.07 (1.01-1.13) |
| Implant/Injection | 1,215 (17.2) | 1.06 (0.99-1.12) |
| N, number; IR, crude incidence rate (per 1,000 person-years); HR, hazard ratio; CI, confidence interval; LNG-IUD – Levonorgestrel-releasing intrauterine device | | |

**Table S6. Use of contraceptives and subsequent risk of suicide in the nationwide population-based cohort.**

| Women | **All women** | | **Without history of depression** | | **With history of depression** | |
| --- | --- | --- | --- | --- | --- | --- |
|  | N (IR) | HR (95% CI) | N (IR) | HR (95% CI) | N (IR) | HR (95% CI) |
| No use | 1,011 (0.1) | 1.0 | 233 (0.0) | 1.0 | 778 (0.6) | 1.0 |
| Oral combined | 72 (0.0) | 0.47 (0.37-0.61) | 34 (0.0) | 0.69 (0.48-1.00) | 38 (0.2) | 0.38 (0.27-0.53) |
| Oral progestin-only | <5 (0.0) | 0.28 (0.10-0.75) | <5 (0.0) | 0.82 (0.26-2.57) | <5 (0.0) | 0.09 (0.01-0.67) |
| Non-oral combined | 47 (0.1) | 0.53 (0.40-0.71) | 14 (0.0) | 0.65 (0.38-1.12) | 33 (0.3) | 0.49 (0.35-0.70) |
| Non-oral progestin-only | 141 (0.1) | 0.88 (0.74-1.05) | 28 (0.0) | 0.91 (0.61-1.35) | 113 (0.4) | 0.87 (0.71-1.06) |

N, number of events; IR, crude incidence rate (per 1,000 person-years); HR, hazard ratio; CI, confidence interval.

^*^ The estimates were adjusted for year of birth, attained age, civil partnership, educational level, household income, parity, history of psychiatric disorder, and additionally history of suicidal behavior in analysis of repeated events

| **Table S7. Use of non-oral progestin-only contraceptives (different routes of administration) and the risk of suicidal behavior among all women in the nationwide population-based cohort, using first event in population analysis.** | | |
| --- | --- | --- |
|  | N (IR) | HR* (95% CI) |
| No use | 33,434 (4.2) | 1.0 |
| **Non-oral progestin-only (Total)** | **6,754 (5.0)** | **1.17 (1.14-1.20)** |
| Gonane (IUD) | 4,058 (4.3) | 1.15 (1.11-1.19) |
| Gonane (implant) | 1,773 (7.3) | 1.33 (1.27-1.40) |
| Pregnane (injection) | 923 (5.3) | 1.02 (0.96-1.09) |

N, number of events; IR, crude incidence rate (per 1,000 person-years); HR, hazard ratio; CI, confidence interval; IUD; Intrauterine device.

^*^ The estimates were adjusted for year of birth, attained age, civil partnership, educational level, household income, parity, history of psychiatric disorder, and additionally history of suicidal behavior in analysis of repeated events.

**Appendix 1**

**Stockholm sub-cohort and depression diagnosis**

In the nationwide cohort, we identified depression through both inpatient care and mental health specialist care according to the Patient Register. The Stockholm population-based cohort is a subsample of the nationwide cohort including all women that resided in Stockholm during the study period (n=475 196, 23·3% of the nationwide cohort). In this cohort, we identified clinical diagnosis of depression not only from the Patient Register but also from the primary care setting. Women with a diagnosis of depression before the start of follow-up were excluded.
