# Supplementary material for: Hormonal contraception and the risk of suicidal behaviour: a Swedish nationwide register-based study
Source: BMJ Open. 2025 Nov 27;15(11):e105694. doi: 10.1136/bmjopen-2025-105694 (PMC12666169; doi:10.1136/bmjopen-2025-105694)
Supplement: online supplemental figure 1 [file bmjopen-15-11-s002.pdf]

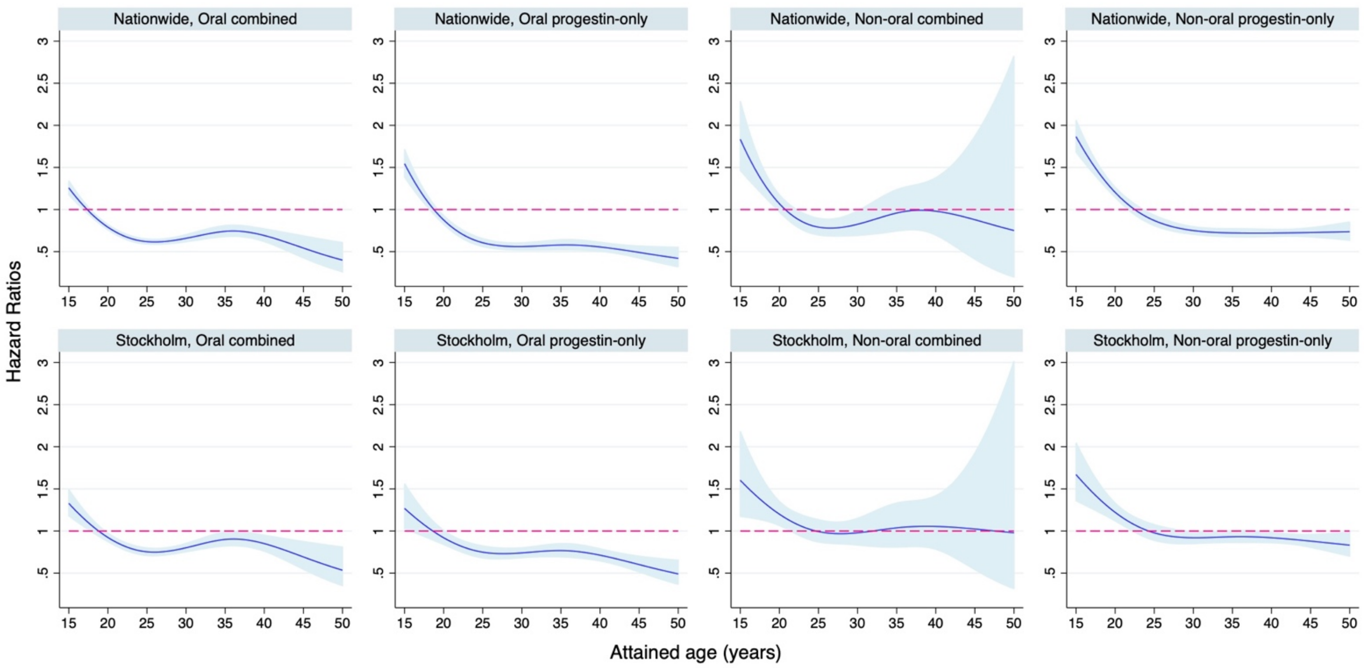

Figure S1. Use of contraceptives and the subsequent risk of Depression diagnosis across attained age in the nationwide population-based cohort and Stockholm population-based cohort, in population analysis. The estimates were adjusted for year of birth, attained age, civil partnership, educational level, household income, parity, and history of psychiatric disorder (other than depression).
