# Supplementary material for: Hormonal contraception and the risk of suicidal behaviour: a Swedish nationwide register-based study
Source: BMJ Open. 2025 Nov 27;15(11):e105694. doi: 10.1136/bmjopen-2025-105694 (PMC12666169; doi:10.1136/bmjopen-2025-105694)
Supplement: Supplementary file 3 [file bmjopen-2025-105694.reviewer_comments.pdf]

PEER REVIEW HISTORY

BMJ Open publishes all reviews undertaken for accepted manuscripts. Reviewers are asked to complete a checklist review form and are provided with free text boxes to elaborate on their assessment. These free text comments are reproduced below.

ARTICLE DETAILS

Title (Provisional)

Hormonal contraception and the risk of suicidal behavior: a Swedish nationwide register-based study

Authors

Aleknaviciute, Jurate; Lu, Donghao; Gotby, Vide; Frans, Emma M; Kuja-Halkola, Ralf; Engberg, Hedvig; Tiemeier, Henning; Lichtenstein, Paul; Fang, Fang; Kushner, Steven; Chang, Zheng

---

VERSION 1 - REVIEW

**Reviewer**	**1**
**Name**	**Toffol, Elena**
**Affiliation**	**University of Helsinki Faculty of Medicine, Department of Public Health**
**Date**	**17-Jun-2025**
**COI**	**None**

I would like to thank the authors for the opportunity of reviewing this interesting and well-written manuscript. In this study the authors examined the associations between use of different types of hormonal contraceptives and the risk of suicidal behavior (attempted suicide and suicide) as well as depression among over two millions women followed up for almost seven years. While the authors found no general increased suicidal risk in relation to the use of oral or non-oral combined hormonal contraceptives, the use of non-oral progestin-only preparations was associated with a higher risk. Also, adolescent girls had higher risk of suicidal behavior in relation to any type of contraceptive used. The findings are of scientific, clinical and public health interest, and add to the existing knowledge on the topic. I have only some, mostly minor questions and comments for the authors:

1. Introduction, page 5, lines 42-43: “Considering that the risk factors for suicidal behavior in adolescence only partially overlap those of adults…” Please, note that not only the risk factors for suicidal behavior do not completely overlap those of adults, but also indications for and types of contraception use are different in this age group. For example, contraceptive

preparations are often prescribed for reasons other than contraception, e.g. for treating conditions such as endometriosis, dysmenorrhea, PCOS… that are themselves related to depression and poorer mental health.

2. Methods, page 7, subchapter Outcomes, line 50: "We defined a suicidal behavior event as suicide attempt or death by suicide": suicide and suicide attempt, and even more (non-suicidal) self-injurious behavior, are distinct behaviors, with different underlying risk factors and prevalence rates in different age groups. Why did the authors decide to combine all of them together? I would like to see estimates for suicide separate from suicidal attempts.

3. Methods, page 8, lines 1-9: Please, make clear already at this stage that cases from Primary Care visits were mostly missing (with the exception of part of the analyses regarding depression). I am not sure about Sweden, but in general almost half of attempted suicide/self-harm cases do not reach health care attention at all, and many of the cases that do get in contact with health care are treated in primary care only. This may have introduced a bias.

4. Methods, page 8, subchapter Covariables: psychiatric history is not mentioned here among the covariables, but it is in fact taken into account in the analyses. Please, add this information. How was history of psychiatric disorders retrieved? Was it based on the Patient Register? Were also Primary care data utilized, and if not, why? How about prescription data (e.g., of antidepressants)?

5. Results, page 11, lines 7-12: "Women using non-oral progestin-only contraceptives had more children and psychiatric disorders compared to women using other types of hormonal contraceptives and non users (Table 1)". I am not sure if the differences were significant, but interesting to note that "women with no use of contraceptives" had the second highest rates of psychiatric disorders (11.1%) and depression (6.8%), after women using non-oral progestin-only contraceptives (11.9% and 7.4%, respectively).

6. Results, page 11: if I understand correctly, the group of non-oral progestin-only contraceptives includes LNG-IUD, etonogestrel implant and DMPA injections. These preparations have different mechanisms of action, some being systemic and other mainly local contraceptives. Thus, the mechanisms how they can affect mood and suicidality are rather different (e.g. although findings support an association between IUD and suicidality/depression, biologically it is hard to explain the association). This point should be addressed in the discussion. Could the authors provide proportions of use of different types of contraceptives in this population and/or in Sweden? What is the proportion of women using DMPA injections, rather than LNG-IUD etc. (same pertains to oral contraceptives) in Sweden? Without this information it is hard to interpret the results.

7. Discussion, page 13, lines 38-40: I think the references “14” and “15” are misplaced: now they refer to the study own results, while they should actually refer to the Danish studies (“studies previously reported associations between hormonal contraception and adverse mental health”).

8. Discussion, page 14, lines 4-12: “Hence, given that use of hormonal contraceptives is a proxy for sexual activity, and younger age of initiation of sexual activity has been associated with elevated rates of risk-taking behavior, self-harm, and adverse health outcomes, the association of hormonal contraceptive use with suicidal behavior may exhibit confounding by indication”. In addition, as mentioned above (please, see my point #1), specific indications for contraceptive prescriptions in adolescents (dysmenorrhea, PCOS, endometriosis) are themselves related to higher risk of depression and suicidality.

9. Discussion, page 14, lines 46-48: “The within-individual comparison was designed to attenuate such unmeasured potential confounders, assuming they are stable across time.” In addition, I think the results of your within-individual analyses support the hypothesis that mood (and related) side effects of hormonal contraception tend to co-occur in a subgroup of (hormonally?) sensitive vulnerable women, that we should aim at identifying for allowing a personalized contraception.

Minor:

1. Methods, page 7, line 55: I think “or self-harm” is mistakenly repeated.

2. Methods, page 10, line 18: “new use and prevalent use of antidepressants”: I think it should be “new use and prevalent use of hormonal contraceptives”, instead.

Reviewer **2**

Name **Stika, Catherine S**

Affiliation **Northwestern University Feinberg School of Medicine, Obstetrics and Gynecology**

Date **09-Jul-2025**

COI **None**

This important manuscript reported the results of a large Swedish nationwide registry study in over 2 million women between 2006 and 2013 which explored the relationships between hormonal contraception and suicide behavior and depression.

The questions that I pose reflect my many years of working as an obstetrician gynecologist, especially in a complex contraception clinic where I cared for women with multiple medical problems for whom non-oral, progestin-only contraception was usually the preferred, if not only, contraceptive option. Because the results of this study will significantly impact our field, I think it is critically important that the authors

address my following concerns.

1. This is a minor, trivial question: I am curious why the authors chose the phrase "…long-lasting reversible forms of contraceptives…" when the more popular phrase in English is "long-acting reversible" (LARC = long-active reversible contraception) (P5, LL53-60)

2. I would encourage this author team to probe further the reasons why their results differed from those found in the two Danish studies which also examined relationships between contraceptives and suicide/depression. Although many of their findings are similar, the fact that combined E/P hormonal was associated with increased risk of depression and suicide in the Danish studies and not in this manuscript has important implications which need to be clarified.

3. Given the incredible availability of data in Scandinavian health registries, I am truly disappointed that the authors did not include other medical morbidities as co-variates. It is not just gynecologic issues that predispose to the use of progestin-only contraceptives. Multiple medical problems (beyond thromboembolic disease and issues of non-compliance) are more safely managed with progestin-only contraceptives, including the important neurological conditions of migraine with aura and epilepsy – both of which are independently associated with increased risks of suicide and depression. Not including these important factors as potential confounding co-variates in their analysis is a critical flaw. Those of us that care for these people need to know if creating a progestin-dominant environment significantly increases the risk of suicide or if the major factor is their underlying medical condition.

4. Progestin-releasing intrauterine devices are appropriately included in the non-oral, progestin-only contraceptives; however, the systemic concentrations of progestin (levonorgestrel) attained with this method are significantly less than those with depo-MPA or progestin implants – and often so low that ovulation itself is not suppressed. Therefore, one could hypothesize that its impact on depression and suicidality might be less. Given that this type of IUD is often employed in the same groups of people with medical co-morbidities predisposing them to increased risks of suicide, it would be interesting (important) to see a sub-analysis separating outcomes between the progestin IUD and other forms of non-oral, progestin-only contraceptives.

**Reviewer**	**3**
**Name**	**Hoffmire, CA**
**Affiliation**	**U.S. Department of Veterans Affairs, Rocky Mountain MIRECC for Suicide Prevention**
**Date**	**24-Jun-2025**
**COI**	**None**

Thank you for the opportunity to review this important population-based cohort study which assessed overall and age-group specific associations between hormonal contraceptive use and suicide in the Swedish population. The findings from this study are important to disseminate in a timely manner. However, there are some concerns to first be addressed to ensure findings are as unbiased and clear as possible.

• The outcome used is a combined suicidal behavior outcome – SA and death. Additionally, SA included self-harm of undetermined intent. While such combined outcomes are justifiable, they have limitations and it is important for large studies such as this to also look at a specific suicide mortality outcome. That should be feasibly and could be done as an additional sensitivity analysis.

• Covariates – psychiatric disorder was not listed in the covariate section but rather later under suicidal behavior. Please list as a covariate for clarity. Additionally, only psychiatric disorders that precede initial contraception prescriptions are true potential confounders. Those that follow are potentially mediators of the relationship between contraception and suicidal behaviors. It is unclear how this was handled and if

there need to be edits to the analysis. It may also be possible to conduct mediation analyses which could be very informative. Additionally, rather than excluding those with prior SA that could be a covariate/confounder adjusted for. It is unclear why those with prior SA were instead included in the main analysis. I

• One of my biggest concerns is the statement in the methods section that only women with at least 1 suicidal behavior event were included in the within-individual analysis. That analysis is very important to addressing unmeasured confounding, as discussed, but it is not clear why this analysis needs to be or should be limited to those with at least 1 suicidal behavior. It is a comparison of individuals exposed and unexposed time periods (based on contraception use) not a comparison of which periods they did and did not have an event in. Please justify approach used and address any undiscussed limitations or revise this analysis (I believe the latter is most appropriate).

• Line 18: "…new use and prevalent use of antidepressants." I believe antidepressants should be revised to hormonal contraceptives.

• Reason for the Stockholm sub-cohort is not fully clarified in lines 41-43, I think there is missing information that needs to briefly be added.

VERSION 1 - AUTHOR RESPONSE

Reviewer: 1

Dr. Elena Toffol, University of Helsinki Faculty of Medicine

I would like to thank the authors for the opportunity of reviewing this interesting and well-written manuscript. In this study the authors examined the associations between use of different types of hormonal contraceptives and the risk of suicidal behavior (attempted suicide and suicide) as well as depression among over two millions women followed up for almost seven years. While the authors found no general increased suicidal risk in relation to the use of oral or non-oral combined hormonal contraceptives, the use of non-oral progestin-only preparations was associated with a higher risk. Also, adolescent girls had higher risk of suicidal behavior in relation to any type of contraceptive used. The findings are of scientific, clinical and public health interest, and add to the existing knowledge on the topic. I have only some, mostly minor questions and comments for the authors:

Response: *We sincerely appreciate the reviewer's endorsement of our study, and the thoughtful suggestions to further improve the manuscript.*

1. Introduction, page 5, lines 42-43: "Considering that the risk factors for suicidal behavior in adolescence only partially overlap those of adults…" Please, note that not only the risk factors for suicidal behavior do not completely overlap those of adults, but also indications for and types of contraception use are different in this age group. For example, contraceptive preparations are often prescribed for reasons other than contraception, e.g. for treating conditions such as endometriosis, dysmenorrhea, PCOS… that are themselves related to depression and poorer mental health.

Response: *We agree with the reviewer's viewpoint and have revised the paragraph accordingly to address this concern (page 5).*

Modified text manuscript: The partial overlap of risk factors for suicidal behavior between adolescents and adults[18] poses a significant question as to whether the link between hormonal contraception and suicidal behavior endures into adulthood. Contraceptive prescriptions in younger age groups often encompass diverse health considerations beyond contraception alone,

underscoring the complexity in the nature of the age-dependent relationship between hormonal contraception, mental health and suicidal behavior.

2. Methods, page 7, subchapter Outcomes, line 50: "We defined a suicidal behavior event as suicide attempt or death by suicide": suicide and suicide attempt, and even more (non-suicidal) self-injurious behavior, are distinct behaviors, with different underlying risk factors and prevalence rates in different age groups. Why did the authors decide to combine all of them together? I would like to see estimates for suicide separate from suicidal attempts.

Response: *We appreciate the insightful feedback regarding our analyses of suicide, attempted suicide, and non-suicidal self-injurious behavior. We agree with the reviewer's suggestion and have now conducted an additional analysis focused exclusively on suicide (**Table A**, below). However, these analyses suffer from small sample sizes and insufficient statistical power, particularly for investigating age-dependent associations. We therefore prefer not to include this table into the manuscript unless the reviewer and editor disagree.*

Table A. Use of contraceptives and subsequent risk of suicide in the nationwide population-based cohort.

Women	**All women**		**Without history of depression**		**With history of depression**	
	N (IR)	HR (95% CI)	N (IR)	HR (95% CI)	N (IR)	HR (95% CI)
No use	1,011 (0.1)	1.0	233 (0.0)	1.0	778 (0.6)	1.0
Oral combined	72 (0.0)	0.47 (0.37-0.61)	34 (0.0)	0.69 (0.48-1.00)	38 (0.2)	0.38 (0.27-0.53)
Oral progestin-only	<5 (0.0)	0.28 (0.10-0.75)	<5 (0.0)	0.82 (0.26-2.57)	<5 (0.0)	0.09 (0.01-0.67)
Non-oral combined	47 (0.1)	0.53 (0.40-0.71)	14 (0.0)	0.65 (0.38-1.12)	33 (0.3)	0.49 (0.35-0.70)
Non-oral progestin-only	141 (0.1)	0.88 (0.74-1.05)	28 (0.0)	0.91 (0.61-1.35)	113 (0.4)	0.87 (0.71-1.06)

Moreover, while suicide, attempted suicide, and (non-suicidal) self-injurious behavior are distinct behaviors, they exhibit strong evidence of shared risk factors, potential escalation within individuals, and interconnectedness within the broader phenomenon of self-harm. For example, non-suicidal self-injurious behavior significantly increases the risk of future suicide attempts. Furthermore, there are considerable challenges in definitively differentiating between suicidal self-harm and non-suicidal self-harm, particularly in large-scale epidemiological studies. Therefore, we reasoned that aggregating these behaviors allowed for a more robust analysis of the overall impact of hormonal contraception on self-harm outcomes across different age groups.

However, we fully acknowledge the need for sensitivity and careful interpretation of analyses using these combined data. Nonetheless, we are confident that this approach yields valuable insights into the complex relationship between hormonal contraception and the complete spectrum of self-harm behaviors, ultimately informing future research and potentially shaping future interventions.

3. Methods, page 8, lines 1-9: Please, make clear already at this stage that cases from Primary Care visits were mostly missing (with the exception of part of the analyses regarding depression). I am not sure about Sweden, but in general almost half of attempted suicide/self-harm cases do not reach health care attention at all, and many of the cases that do get in contact with health care are treated in primary care only. This may have introduced a bias.

Response: *We appreciate the reviewer's concern about missing data from primary care visits and the potential for introducing a bias towards more severe cases. We acknowledge the need for future research using more comprehensive data that includes primary care for a complete picture and have now modified the manuscript text accordingly (pages 7-8). We have also acknowledged this in the limitation on Pages 16-17.*

Modified text manuscript: The Patient Register includes nationwide information on hospital discharge records since 1987 and outpatient visits to specialty care since 2001. However, it is important to note that information on cases arising from primary care visits is largely missing. In contrast, the Causes of Death Register covers over 99% of deaths occurring in Sweden.

4. Methods, page 8, subchapter Covariables: psychiatric history is not mentioned here among the covariables, but it is in fact taken into account in the analyses. Please, add this information. How was the history of psychiatric disorders retrieved? Was it based on the Patient Register? Were also Primary care data utilized, and if not, why? How about prescription data (e.g., of antidepressants)?

Response: *We appreciate the reviewer's comment on this important dimension to the analyses, and have now better clarified the source of the psychiatric history data used in the study. This information has been added to the methodology section on page 8, outlining that the data were indeed obtained from the Patient Register.*

Modified text manuscript: Parity data were extracted from the Medical Birth Register and Multi-Generation Register. History of psychiatric disorders and suicidal behavior were obtained from the Patient Register.

5. Results, page 11, lines 7-12: "Women using non-oral progestin-only contraceptives had more children and psychiatric disorders compared to women using other types of hormonal contraceptives and non users (Table 1)". I am not sure if the differences were significant, but interesting to note that "women with no use of contraceptives" had the second highest rates of psychiatric disorders (11.1%) and depression (6.8%), after women using non-oral progestin-only contraceptives (11.9% and 7.4%, respectively).

Response: *We appreciate the reviewer highlighting this intriguing result. The finding that "no use of contraceptives" was associated with the second-highest rates of history of psychiatric disorders and depression, closely following non-oral progestin-only users, indeed underscores the complexity of the relationship between contraception and mental health. While counterintuitive at first glance, it suggests the potential influence of factors beyond contraceptive method alone. Given that the highlighted groups are comparable by age, civil status, and parity, we acknowledge the need for*

further exploration of additional factors including socioeconomic status, healthcare access, and underlying health conditions that could illuminate these patterns.

6. Results, page 11: if I understand correctly, the group of non-oral progestin-only contraceptives includes LNG-IUD, etonogestrel implant and DMPA injections. These preparations have different mechanisms of action, some being systemic and other mainly local contraceptives. Thus, the mechanisms how they can affect mood and suicidality are rather different (e.g. although findings support an association between IUD and suicidality/depression, biologically it is hard to explain the association). This point should be addressed in the discussion. Could the authors provide proportions of use of different types of contraceptives in this population and/or in Sweden? What is the proportion of women using DMPA injections, rather than LNG-IUD etc. (same pertains to oral contraceptives) in Sweden? Without this information it is hard to interpret the results.

Response: *We appreciate the reviewer's point about the heterogeneity within the 'non-oral progestin-only contraceptives' group. Combining methods like the LNG-IUD, etonogestrel implant, and DMPA injections, which have diverse mechanisms of action, limits our ability to draw specific conclusions about their individual effects on mood and suicidality. Furthermore, the dominance of the LNG-IUD group within this category adds to the challenge of isolating potential effects of other methods (**Table B**, below). While our findings suggest an association within this group, future research differentiating between these methods and exploring the distinct biological mechanisms of each method and their potential link to mood and suicidality is critical to provide a more comprehensive understanding of their potential impact on mental health outcomes.*

Table B. Use of non-oral progestin-only contraceptives (different routes of administration) and the risk of suicidal behavior among all women in the nationwide population-based cohort, using first event in population analysis.

	N	(IR)	HR (95% CI)
No use	33,434	(4.2)	1.0
**Non-oral progestin-only (Total)**	**6,754**	**(5.0)**	**1.17 (1.14-1.20)**
Gonane (IUD)	4,058	(4.3)	1.15 (1.11-1.19)
Gonane (implant)	1,773	(7.3)	1.33 (1.27-1.40)
Pregnane (injection)	923	(5.3)	1.02 (0.96-1.09)

7. Discussion, page 13, lines 38-40: I think the references “14” and “15” are misplaced: now they refer to the study own results, while they should actually refer to the Danish studies (“studies previously reported associations between hormonal contraception and adverse mental health”).

Response: *We thank the reviewer for the careful reading. This was indeed a mix-up of the references, which has now been corrected in the revised manuscript.*

8. Discussion, page 14, lines 4-12: “Hence, given that use of hormonal contraceptives is a proxy for sexual activity, and younger age of initiation of sexual activity has been associated with elevated rates of risk-taking behavior, self-harm, and adverse health outcomes, the association of hormonal contraceptive use with suicidal behavior may exhibit confounding by indication”. In addition, as mentioned above (please, see my point #1), specific indications for contraceptive prescriptions in

adolescents (dysmenorrhea, PCOS, endometriosis) are themselves related to higher risk of depression and suicidality.

Response: *We fully agree and have now modified the manuscript text on page 14 to address this point.*

Modified text manuscript: Furthermore, specific indications for contraceptive prescriptions in adolescents, such as dysmenorrhea, polycystic ovary syndrome, and endometriosis, are associated with a higher risk of depression and suicidality.

9. Discussion, page 14, lines 46-48: “The within-individual comparison was designed to attenuate such unmeasured potential confounders, assuming they are stable across time.” In addition, I think the results of your within-individual analyses support the hypothesis that mood (and related) side effects of hormonal contraception tend to co-occur in a subgroup of (hormonally?) sensitive vulnerable women, that we should aim at identifying for allowing a personalized contraception.

Response: *We appreciate the reviewer’s highlighting the strength of our within-individual design, which mitigates unmeasured confounders and offers insight into individual associations between hormonal contraception and mood changes. However, while we share the view proposed by the reviewer, we feel that further investigation would be needed to confirm the hypothesis of a "subgroup of hormonally sensitive vulnerable women", which is difficult in the current dataset due to limitations of the cohort (sample size, potential time-varying confounders, complex mood influences). Moreover, we fully agree with the reviewer about the transformative potential of personalized contraception. Our perspective is that larger, more diverse samples with longer follow-up periods and more sophisticated methods will be crucial to translate these and related findings into an evidence-based approach to personalized contraception guided by a thorough understanding of the underlying biology, clinically reliable biomarkers, and careful consideration of ethical issues.*

Modified text manuscript: Further research is needed crucial to translate these and related findings into an evidence-based approach to personalized contraception guided by a thorough understanding of the underlying biology, clinically reliable biomarkers, and careful consideration of ethical issues.

Minor:

1. Methods, page 7, line 55: I think “or self-harm” is mistakenly repeated.

2. Methods, page 10, line 18: “new use and prevalent use of antidepressants”: I think it should be “new use and prevalent use of hormonal contraceptives”, instead.

Response: *We thank the reviewer for noticing these mistakes. We have now corrected them the manuscript text.*

Reviewer: 2

Dr. Catherine S Stika, Northwestern University Feinberg School of Medicine

This important manuscript reported the results of a large Swedish nationwide registry study in over 2 million women between 2006 and 2013 which explored the relationships between hormonal contraception and suicide behavior and depression.

The questions that I pose reflect my many years of working as an obstetrician gynecologist, especially in a complex contraception clinic where I cared for women with multiple medical problems for whom non-oral, progestin-only contraception was usually the preferred, if not only, contraceptive option. Because the results of this study will significantly impact our field, I think it is critically important that the authors address my following concerns.

Response: *We thank the reviewer for the support of our study and the opportunity to address these important concerns.*

1. This is a minor, trivial question: I am curious why the authors chose the phrase "…long-lasting reversible forms of contraceptives…" when the more popular phrase in English is "long-acting reversible" (LARC = long-active reversible contraception) (P5, LL53-60)

Response: *We appreciate the reviewer highlighting the advantages of using "long-acting reversible contraception (LARC)." We fully endorse adopting this term as it accurately conveys the key characteristics of these methods and changed the text accordingly.*

Modified text manuscript: Moreover, although a wide range of different types of hormonal contraceptives are available, there has been an increasing preference for long-acting reversible contraception, for which only limited data is available regarding non-oral hormonal contraceptive use and potential adverse effects on mental health outcomes.[6,14-16]

2. I would encourage this author team to probe further the reasons why their results differed from those found in the two Danish studies which also examined relationships between contraceptives and suicide/depression. Although many of their findings are similar, the fact that combined E/P hormonal was associated with increased risk of depression and suicide in the Danish studies and not in this manuscript has important implications which need to be clarified.

Response: *Thank you for raising this important question. Unlike previous studies, including the Danish studies, that focused on new users and younger women, we included both "never users" and "former users" in our reference group. This broader approach allowed us to explore the association for all women, regardless of their contraceptive history, highlighting its public health significance given the widespread contraceptive use across all ages. While it was more challenging to also identify older women who never used contraceptives, we believe that including them in our study enhances the robustness and applicability of the findings.*

The earlier Danish studies limited their cohort to younger women, among whom associations were reported between hormonal contraception and suicidal behavior. However, it remains unclear if this persists among older women, a group with significant contraceptive use and higher suicide rates. Our study contributes to filling this gap, thereby informing public health strategies related to women's reproductive health and mental well-being across the lifespan.

*We acknowledge the potential issue of healthy user bias, in which women who initiate or continue hormonal contraception might be generally healthier. To address this, we conducted sensitivity analyses using different definitions for "exposed" and "non-exposed" groups (**Table C**, below). Notably, these analyses further confirmed the main findings of our study, suggesting minimal influence from potential bias.*

While further research using longitudinal designs and larger, more diverse populations is needed to definitively address this bias, our findings offer novel insights into this complex relationship. In particular, they highlight the importance of considering the full spectrum of reproductive ages when evaluating the potential impact of hormonal contraception on mental health.

Table C. Use of hormonal contraceptives and the risk of suicidal behavior among all women, using first event population analyses: i) Main analysis; ii) Sensitivity analysis by extending contraceptive prescription by four weeks, and iii) Additional sensitivity analysis of redefined exposed groups (new users, prevalent users).

	Main Analysis		*Extended Rx (4 wks)*		*Redefined Exposed Periods*			
	Users		Users		New Users		Prevalent Users	
	N (IR)	HR (95% CI)	N (IR)	HR (95% CI)	N (IR)	HR (95% CI)	N(IR)	HR (95% CI)
No use	33 434 (4·2)	1·0	32 522 (4·2)	1·0	29 247 (4·3)	1·0	29 247 (4·3)	1·0
Oral combined	6 020 (3·3)	0·82 (0·79-0·84)	6 436 (3·4)	0·84 (0·82-0·87)	2 569 (3·1)	0·76 (0·73-0·80)	2 732 (3·7)	0·87 (0·83-0·90)
Oral progestin-only	3 154 (3·6)	0·88 (0·85-0·92)	3 447 (3·7)	0·92 (0·88-0·95)	1 172 (3·4)	0·87 (0·82-0·93)	1 716 (3·8)	0·89 (0·85-0·94)
Non-oral combined	569 (3·8)	0·91 (0·84-0·99)	653 (4·1)	0·97 (0·90-1·05)	132 (3·7)	0·88 (0·74-1·05)	390 (3·9)	0·90 (0·81-1·00)
Non-oral progestin-only	6 754 (5·0)	1·17 (1·14-1·20)	6 873 (5·0)	1·18 (1·15-1·21)	4 163 (4·7)	1·16 (1·12-1·20)	2 234 (5·9)	1·21 (1·16-1·26)

N, number of events; IR, crude incidence rate (per 1,000 person-years); HR, hazard ratio; CI, confidence interval. Adjusted for year of birth, attained age, civil partnership, educational level, household income, parity, history of psychiatric disorder.

3. Given the incredible availability of data in Scandinavian health registries, I am truly disappointed that the authors did not include other medical morbidities as co-variates. It is not just gynecologic issues that predispose to the use of progestin-only contraceptives. Multiple medical problems (beyond thromboembolic disease and issues of non-compliance) are more safely managed with progestin-only contraceptives, including the important neurological conditions of migraine with aura and epilepsy – both of which are independently associated with increased risks of suicide and depression. Not including these important factors as potential confounding co-variates in their analysis is a critical flaw. Those of us that care for these people need to know if creating a progestin-dominant environment significantly increases the risk of suicide or if the major factor is their underlying medical condition.

Response: *We appreciate the reviewer's valuable point regarding the limited covariates, particularly for conditions like migraine with aura and epilepsy. Expanding the scope would have undoubtedly provided a richer, more nuanced picture. However, we aimed to mitigate these limitations through within-individual analyses. By comparing risk and control periods within the same individual, we could partially control for chronic comorbidities like migraine or epilepsy. This approach strengthens our understanding of the association between changes in progestin exposure and suicide risk, even though pre-existing health isn't fully accounted for. The hypothesis raised by the reviewer is*

undoubtedly an important one that we and others would like to focus on addressing in a future manuscript specifically dedicated to this question.

4. Progestin-releasing intrauterine devices are appropriately included in the non-oral, progestin-only contraceptives; however, the systemic concentrations of progestin (levonorgestrel) attained with this method are significantly less than those with depo-MPA or progestin implants – and often so low that ovulation itself is not suppressed. Therefore, one could hypothesize that its impact on depression and suicidality might be less. Given that this type of IUD is often employed in the same groups of people with medical co-morbidities predisposing them to increased risks of suicide, it would be interesting (important) to see a sub-analysis separating outcomes between the progestin IUD and other forms of non-oral, progestin-only contraceptives.

Response: *We appreciate the reviewer's point about the heterogeneity among the non-oral progestin-only contraceptives group. As recommended by the reviewer, we have now conducted the proposed sensitivity analysis (**Table B**, below). The findings not only validate the association of gonane-containing IUDs with suicidal behavior, but also confirm the reviewer's hypothesis that gonane-containing implants have an even greater risk increase. In contrast, pregnane-based injections were not associated with an increased risk. However, future research differentiating between these methods and exploring the distinct biological mechanisms of each method and their potential link to mood and suicidality should be performed to provide a more comprehensive understanding of their potential impact on mental health outcomes. Such analyses could provide more nuanced insights for informing clinical decision-making.*

Table B. Use of non-oral progestin-only contraceptives (different routes of administration) and the risk of suicidal behavior among all women in the nationwide population-based cohort, using first event in population analysis.

	N	(IR)	HR (95% CI)
No use	33,434	(4.2)	1.0
**Non-oral progestin-only (Total)**	**6,754**	**(5.0)**	**1.17 (1.14-1.20)**
Gonane (IUD)	4,058	(4.3)	1.15 (1.11-1.19)
Gonane (implant)	1,773	(7.3)	1.33 (1.27-1.40)
Pregnane (injection)	923	(5.3)	1.02 (0.96-1.09)

Reviewer: 3

Dr. CA Hoffmire, U.S. Department of Veterans Affairs

Thank you for the opportunity to review this important population-based cohort study which assessed overall and age-group specific associations between hormonal contraceptive use and suicide in the Swedish population. The findings from this study are important to disseminate in a timely manner. However, there are some concerns to first be addressed to ensure findings are as unbiased and clear as possible.

Response: *We appreciate reviewer's support and the opportunity to address these important concerns.*

• The outcome used is a combined suicidal behavior outcome – SA and death. Additionally, SA included self-harm of undetermined intent. While such combined outcomes are justifiable, they have limitations and it is important for large studies such as this to also look at a specific suicide mortality outcome. That should be feasibly and could be done as an additional sensitivity analysis.

Response: *We appreciate the insightful feedback regarding our analyses of suicide attempt and death. We agree with the reviewer's suggestion and have now conducted an additional analysis focused exclusively on suicide (**Table A**, below). However, these analyses suffer from small sample sizes and insufficient statistical power, particularly for investigating age-dependent associations.*

Table A. Use of contraceptives and subsequent risk of suicide in the nationwide population-based cohort.

Women	All women		Without history of depression		With history of depression	
	N (IR)	HR (95% CI)	N (IR)	HR (95% CI)	N (IR)	HR (95% CI)
No use	1,011 (0.1)	1.0	233 (0.0)	1.0	778 (0.6)	1.0
Oral combined	72 (0.0)	0.47 (0.37-0.61)	34 (0.0)	0.69 (0.48-1.00)	38 (0.2)	0.38 (0.27-0.53)
Oral progestin-only	<5 (0.0)	0.28 (0.10-0.75)	<5 (0.0)	0.82 (0.26-2.57)	<5 (0.0)	0.09 (0.01-0.67)
Non-oral combined	47 (0.1)	0.53 (0.40-0.71)	14 (0.0)	0.65 (0.38-1.12)	33 (0.3)	0.49 (0.35-0.70)
Non-oral progestin-only	141 (0.1)	0.88 (0.74-1.05)	28 (0.0)	0.91 (0.61-1.35)	113 (0.4)	0.87 (0.71-1.06)

Moreover, while suicide, attempted suicide, and (non-suicidal) self-injurious behavior are distinct behaviors, they exhibit strong evidence of shared risk factors, potential escalation within individuals, and interconnectedness within the broader phenomenon of self-harm. For example, non-suicidal self-injurious behavior significantly increases the risk of future suicide attempts. Furthermore, there are considerable challenges in definitively differentiating between suicidal self-harm and non-suicidal self-harm, particularly in large-scale epidemiological studies. Therefore, we reasoned that aggregating these behaviors allowed for a more robust analysis of the overall impact of hormonal contraception on self-harm outcomes across different age groups.

However, we fully acknowledge the need for sensitivity and careful interpretation of analyses using these combined data. Nonetheless, we are confident that this approach yields valuable insights into the complex relationship between hormonal contraception and the complete spectrum of self-harm behaviors, ultimately informing future research and potentially shaping future interventions.

• Covariates – psychiatric disorder was not listed in the covariate section but rather later under suicidal behavior. Please list as a covariate for clarity. Additionally, only psychiatric disorders that precede initial contraception prescriptions are true potential confounders. Those that follow are potentially mediators of the relationship between contraception and suicidal behaviors. It is unclear how this was handled and if there need to be edits to the analysis. It may also be possible to conduct

mediation analyses which could be very informative. Additionally, rather than excluding those with prior SA that could be a covariate/confounder adjusted for. It is unclear why those with prior SA were instead included in the main analysis.

Response: *Thank you for highlighting these concerns. Psychiatric disorders as covariates are now indeed included in the covariables section (page 8).*

Modified text manuscript: Parity was extracted using the Medical Birth Register and Multi-Generation Register. History of psychiatric disorder, and history of suicidal behavior data was acquired from the Patient Register. Mean individualized household income was binned into quartiles throughout the follow-up period.

When considering mediation analysis, the delayed and sometimes inaccurate diagnosis of psychiatric conditions presents a challenge. Such diagnostic delays complicate determination of whether a diagnosis precedes (as potential confounders) or follows (as potential mediators) the use of hormonal contraception (HC). This ambiguity regarding the temporal relationship of psychiatric diagnoses and HC usage has the potential to hinder efforts to address confounding effectively. Furthermore, in the first event analysis, women with suicidal behavior events before the start of follow-up were excluded.

• One of my biggest concerns is the statement in the methods section that only women with at least 1 suicidal behavior event were included in the within-individual analysis. That analysis is very important to addressing unmeasured confounding, as discussed, but it is not clear why this analysis needs to be or should be limited to those with at least 1 suicidal behavior. It is a comparison of individuals exposed and unexposed time periods (based on contraception use) not a comparison of which periods they did and did not have an event in. Please justify approach used and address any undiscussed limitations or revise this analysis (I believe the latter is most appropriate).

Response: *You raise a valid point regarding the inclusion criteria for the within-individual analysis. While the chosen method, analyzes pairs or sets within individuals where exposure and outcome differ (discordant) [Petersen et al., The BMJ. (2016), 12(354) :i4515],we agree that limiting the analysis to those with at least one suicidal behavior event does warrant a clarification. Fixed-effects models rely on within-person variation both to identify the effect of a time-varying exposure while limiting potential confounding by measured and unmeasured time-invariant factors and to account for correlations that arise between repeated measures [Keen et al., JAMA Pediatrics (2023), 177(8): 818-826]. Inclusion criteria are part of the design of the fixed effects regression model, in which those who were never exposed cannot contribute to analyses of change of outcome, and are thus dropped from the model. Conversely, all individuals who initiated contraceptive use during the observation period were included in the analysis. We have now modified the manuscript text accordingly to clarify the model design (pages 9-10).*

Modified text manuscript (page 9): To better control for unmeasured confounders, we conducted a within-individual analysis using fixed-term regression. Fixed-effects models rely on within-person variation both on the exposure and outcome to identify the effect of a time-varying exposure while limiting potential confounding by measured and unmeasured time-invariant factors and to account for correlations that arise between repeated measures [Keen et al., *JAMA Pediatrics* (2023), 177(8): 818-826].

Modified text manuscript (page 10): Individuals who never used contraceptives, however, cannot contribute to analyses of exposure changes in contraceptive use. Moreover, individuals who started using contraceptives at any point during the study period were included in the analysis.

• Line 18: "…new use and prevalent use of antidepressants." I believe antidepressants should be revised to hormonal contraceptives.

Response: *We thank the reviewer for noticing this mistake. We have now corrected it in the main text.*

• Reason for the Stockholm sub-cohort is not fully clarified in lines 41-43, I think there is missing information that needs to briefly be added.

Response: *We fully agree. The Stockholm sub-cohort analysis allowed us to capture a more comprehensive picture of depression diagnoses by including data not only from the Patient Register, but also from the primary care setting. This clarification has now been added to the manuscript text (page 11).*

Modified text manuscript: We examined associations between hormonal contraceptive use and clinical diagnoses of depression using both the nationwide cohort, as described for the analysis of suicidal behavior, and an independent Stockholm population-based cohort which we were able to complement with depression diagnoses from the Primary Care Register (Appendix 1 in the Supplement).

VERSION 2 - REVIEW

**Reviewer**	**1**
**Name**	**Toffol, Elena**
**Affiliation**	**University of Helsinki Faculty of Medicine, Department of Public Health**
**Date**	**17-Jun-2025**
**COI**	

I would like to thank the authors for thoroughly addressing my comments and questions. I only have a few additional questions to the authors:

1. If I understand correctly, women were followed-up during the period 2006-2013. I am wondering if contraceptive trends and/or guidelines have changed in Sweden since then, if different types of HC have been introduced or are nowadays more often used than they were 10 or 20 years ago, and what does this mean in terms of current public health impact of the results (for example, have intrauterine devices with lower doses of hormones been introduced, or has their use become more common? If so, could it be that lower dose IUD may be related to lower suicidal risk than higher dose IUD? Has there been any trend for more women to use lower-dose IUD even at younger age?) I think these points should be addressed in the Discussion/limitations.

2. Methods, page 8, subchapter Outcomes, lines 12-15: "We considered depression as a secondary

outcome, which was defined as a clinical diagnosis of depression obtained from the Patient Register using ICD-10 codes F32-F33." For the sake of clarity, please, clarify here that these were diagnoses recorded during the follow-up period.

3. Methods, page 9, subchapter Statistical analysis, line 14: the authors mention psychiatric history as a variable used for adjustment in regression models. I am wondering if the authors have considered the possible moderation effect of psychiatric history/history of suicidal behavior. There is quite some evidence that women with psychiatric disorders (possibly, but not limited to, related to reproductive events) are more susceptible to mood side effects of contraceptives. Would this be valid also in relation to suicidal behavior? The authors have tested this hypothesis in Table A in the response letter, but not in other (main) analyses.

4. Results, page 12, subchapter "Sensitivity analyses", line 55, "Evaluation of the subsample with no history of contraceptive use before cohort entry": I suggest reminding the reader here that these were women aged 15-18 years.

5. Regarding my comment #2 in first revision: I thank the authors for considering analyses by specific suicide outcome, and appreciate Table A in the response letter. While I agree with the limited power and possibly with the authors' decision not to include the table in the manuscript, I think the approach of combining different suicidal behaviors into one category should be addressed in the Discussion and acknowledged as a partial limitation to a full interpretation of the study findings.

6. Similarly, with regard to my comment #6 in first revision: I appreciate the authors' response and analyses as reported in Table B, but I think that this point (combining different preparations with different local and systemic mechanisms and effects) need to be mentioned and acknowledged in the limitations. Also, do the authors know the proportions of women using different LARC preparations during the study period compared to current trends? This information would help to put the results into context.

Reviewer **2**

Name **Stika, Catherine S**

Affiliation **Northwestern University Feinberg School of Medicine, Obstetrics and Gynecology**

Date **09-Jul-2025**

COI

Both Reviewer #1 and I raised similar concerns regarding the heterogeneity of the various non-oral progestin-only contraceptives and the fact that they were lumped together in the analysis. The authors responded to our concerns with a thorough explanation, including a table showing a breakdown of both numbers of participants and their suicide risks by type of non-oral progestin-only contraceptive, which I found very informative. They also included the following statement to my question:
The findings not only validate the association of gonane-containing IUDs with suicidal behavior, but also confirm the reviewer's hypothesis that gonane-containing implants have an even greater risk increase. In contrast, pregnane-based injections were not associated with an increased risk. However, future research differentiating between these methods and exploring the distinct biological mechanisms of each method and their potential link to mood and suicidality should be performed to provide a more comprehensive understanding of their potential impact on mental health outcomes. Such analyses could provide more nuanced insights for informing clinical decision-making.
And this response to Reviewer #1:
We appreciate the reviewer's point about the heterogeneity within the 'non-oral progestin-only contraceptives' group. Combining methods like the LNG-IUD, etonogestrel implant, and DMPA injections, which have diverse mechanisms of action, limits our ability to draw specific conclusions about their individual effects on mood and suicidality. Furthermore, the dominance of the LNG-IUD group within this

category adds to the challenge of isolating potential effects of other methods (Table B, below). While our findings suggest an association within this group, future research differentiating between these methods and exploring the distinct biological mechanisms of each method and their potential link to mood and suicidality is critical to provide a more comprehensive understanding of their potential impact on mental health outcomes.

However, neither the table nor any part of their response was included in their edited resubmission (Results/Discussion/Limitations or Future Research). Given that two of the three reviewers expressed concerns about this particular issue, I am surprised that the authors chose not to address it in some way within their resubmission. Because I can predict that many readers of this paper will share a similar concern, I strongly encourage the authors to reconsider their response.

Reviewer 3

Name Hoffmire, CA

Affiliation U.S. Department of Veterans Affairs, Rocky Mountain MIRECC for Suicide Prevention

Date 24-Jun-2025

COI

I appreciate the opportunity to re-review this manuscript and the authors efforts to address initial reviewer concerns. However, while some improvement was made to the manuscript, there appears to be a great deal more information in the response to reviewers document that can and should be incorporated into the manuscript itself.

Further, the authors declined to make some key changes suggested by the reviewers. The following concerns remain:

1. Two reviewers brought up notable concerns about the combined outcome. The authors did run a helpful sensitivity analysis for reviewers but chose not to include this in the manuscript itself due to concerns around sample size. Indeed, there are less than 5 suicide outcomes in the oral progestin-only group, and those findings should be suppressed due to unreliability and potential privacy concerns. However, all other groups have a sufficient number of outcomes to report reliable results. Of not this is particularly true for the non-oral progestin only group, for which the results changed (from a significant elevated risk to no association). Failing to report these results and instead stating confidence in the combined outcome is misleading. They should be added as a key sensitivity analysis and potential reasons for the observed differences warrant discussion.

2. Reviewers added not that information on cases resulting from primary care visits is largely missing, but failed to discuss potential implications of that missing data – please add this discussion.

3. A brief mention was added stating that psychiatric disorders were obtained from the patient register. Please add additional information what diagnoses/disorders were captured.

4. One reviewer made an important comment about limited covariates (migraine, epilepsy). The authors responded to the reviewer but failed to add anything about this concern to the discussion as a limitation and/or other consideration – this should certainly be added.

5. The authors response about psychiatric covariates (i.e., regarding confounding vs. effect modification) is insufficient and not compelling or accurate. While it is certainly true that psychiatric diagnoses can be delayed and this does add complication to parsing out confounding and mediation it is very important to try to do so and widely done across the literature evaluating risk factors for suicide.

VERSION 2 - AUTHOR RESPONSE

Reviewer: 1

Dr. Elena Toffol, University of Helsinki Faculty of Medicine

1. If I understand correctly, women were followed-up during the period 2006-2013. I am wondering if contraceptive trends and/or guidelines have changed in Sweden since then, if different types of HC have been introduced or are nowadays more often used than they were 10 or 20 years ago, and what does this mean in terms of current public health impact of the results (for example, have intrauterine devices with lower doses of hormones been introduced, or has their use become more common? If so, could it be that lower dose IUD may be related to lower suicidal risk than higher dose IUD? Has there been any trend for more women to use lower-dose IUD even at younger age?) I think these points should be addressed in the Discussion/limitations.

Response*: We appreciate the insightful questions regarding recent trends and updated guidelines for hormonal contraceptives in Sweden since our study period (2006-2013). Reviewer rightly highlight the evolving landscape of contraceptive methods and their potential impact on public health.*

Since 2013, there have been new formulations introduced such as lower-dose IUDs, which necessitate further investigation. These additional contraceptive options may have different risk profiles compared to prior formulations. Analyzing more recent data and exploring the effects of these methods on mental health, particularly among younger users who may be more likely to choose them, could provide valuable insights.

Accordingly, we acknowledge that our findings in this study may not fully reflect the current dynamics of hormonal contraceptive use and its potential impact on suicidal behavior. Future research focusing on more recent data and the effects of newer methods, including lower-dose IUDs, will be crucial to inform clinical practice and guidelines moving forward.

Modified text manuscript (Discussion, Limitations section, page 18):

We also acknowledge that our findings may not fully reflect evolving demographic trends of hormonal contraceptive use and their potential relationship with suicidal behavior. Future research focusing on recently approved formulations, including lower-dose IUDs, will be crucial to further inform clinical practice and evidence-based guidelines.

2. Methods, page 8, subchapter Outcomes, lines 12-15: “We considered depression as a secondary outcome, which was defined as a clinical diagnosis of depression obtained from the Patient Register using ICD-10 codes F32-F33.” For the sake of clarity, please, clarify here that these were diagnoses recorded during the follow-up period.

Response*: We thank the reviewer for this remark. Accordingly, we have now modified the sentence to clarify that the diagnoses were recorded during the follow-up period.*

Modified text manuscript (Methods, pages 8):

Depression was considered a secondary outcome, operationally defined based on a clinical diagnosis of ICD-10 codes F32-F33 recorded in the Patient Register during the follow-up period.

3. Methods, page 9, subchapter Statistical analysis, line 14: the authors mention psychiatric history as a variable used for adjustment in regression models. I am wondering if the authors have considered the possible moderation effect of psychiatric history/history of suicidal behavior. There is quite some evidence that women with psychiatric disorders (possibly, but not limited to, related to reproductive events) are more susceptible to mood side effects of contraceptives. Would this be valid also in relation to suicidal behavior? The authors have tested this hypothesis in Table A in the response letter, but not in other (main) analyses.

Response*: We appreciate the reviewer's insightful comment regarding the potential moderating effect of psychiatric history or a history of suicidal behavior on the relationship between contraceptive use and mood-related side effects, including suicidal behavior. We did examine this possibility by conducting an analysis comparing outcomes in women with versus without a history of depression. No significant differences in outcomes related to suicidal behavior were observed. In accordance with the reviewer's suggestion, we have now added this analysis and results to the manuscript and as a supplementary table (Table S5).*

Table S5. Use of contraceptives and the subsequent risk of suicide behavior in the nationwide population-based cohort, using first event in population analysis. HRs in population analysis were derived from Cox proportional hazards model using calendar year as underlying time scale, and adjusted for birth year (every 10 years during 1956-1985, and 1986-1998), attained age, marriage status (married, unmarried, or unknown), education level (primary school, high school, college+, or unknown), individualized household income (four quartiles or unknown), parity (0, 1, 2, or 3+), and history of mental disorder (yes or no),, and accounted for within-individual correlations using robust variance estimates.

	N (IR)	HR (95% CI)
Without history of depression		
No use	18,640 (2.8)	1.0
Oral combined	3,927 (2.4)	0.86 (0.83-0.90)
Non-oral combined	335 (2.6)	0.96 (0.86-1.08)
Oral progestin alone	1,976 (2.6)	0.95 (0.91-1.00)
Non-oral progestin alone		
LNG-IUD	2,373 (3.1)	1.18 (1.13-1.24)
Implant/Injection	1,478 (4.3)	1.30 (1.24-1.38)
With history of depression		
No use	14,773 (12.2)	1.0
Oral combined	2,094 (13.0)	0.77 (0.74-0.81)
Non-oral combined	232 (12.3)	0.79 (0.70-0.91)
Oral progestin alone	1,164 (10.2)	0.78 (0.73-0.83)
Non-oral progestin alone		
LNG-IUD	1,724 (10.3)	1.07 (1.01-1.13)
Implant/Injection	1,215 (17.2)	1.06 (0.99-1.12)

N, number; IR, crude incidence rate (per 1,000 person-years); HR, hazard ratio; CI, confidence interval.

4. Results, page 12, subchapter "Sensitivity analyses", line 55, "Evaluation of the subsample with no history of contraceptive use before cohort entry": I suggest reminding the reader here that these were women aged 15-18 years.

Response*: We appreciate this suggestion, and have better clarified the age of the women in the subsample.*

Modified text manuscript (Results, page 13):

Evaluation of the subsample of women aged 15–18 years with no history of contraceptive use before cohort entry yielded similar findings as the main analysis in adolescents (Table S2).

5. Regarding my comment #2 in first revision: I thank the authors for considering analyses by specific suicide outcome and appreciate Table A in the response letter. While I agree with the limited power and possibly with the authors' decision not to include the table in the manuscript, I think the approach of combining different suicidal behaviors into one category should be addressed in the Discussion and acknowledged as a partial limitation to a full interpretation of the study findings.

***Response**: We agree with the reviewer's suggestion. Indeed, combining different types of suicidal behaviors into a single category may have limitations for interpreting our findings. This is now acknowledged as a limitation in the Discussion section of the manuscript.*

Modified text manuscript (Discussion, page 18):

Finally, we note that combining different types of suicidal behaviors into a single category may have limited the ability to fully understand the nuanced relationship between hormonal contraceptive use and suicide risk.

6. Similarly, with regard to my comment #6 in first revision: I appreciate the authors' response and analyses as reported in Table B, but I think that this point (combining different preparations with different local and systemic mechanisms and effects) need to be mentioned and acknowledged in the limitations. Also, do the authors know the proportions of women using different LARC preparations during the study period compared to current trends? This information would help to put the results into context.

***Response**: We appreciate this suggestion and included this point in the Discussion. Unfortunately, we have not performed analyses using more recent data to examine whether the proportion of women using different LARC preparations has evolved over time.*

Modified text manuscript (Discussion, page 17-18):

Additionally, while we categorized contraceptives based on their hormonal composition and administration routes, considerable variability exists within these categories, potentially influencing the interpretation of their association with mental health outcomes. Although the study was not designed and powered to examine each distinct subclass of non-oral progestin-only contraceptive, our preliminary analyses confirmed the association between gonane-containing IUDs and suicidal behavior (Table S7), suggesting that gonane-containing implants may carry an even greater risk of suicidal behavior than other non-oral progestin-only contraceptive subclasses. In contrast, compared to gonane-containing implants, pregnane-based injections appear to be associated with a relatively lower risk of suicidal behavior. Future research should focus on exploring distinct physiological mechanisms of contraceptive subclasses and their relationship with suicidality. Such studies could provide a more comprehensive perspective on mental health outcomes and yield nuanced insights to inform clinical decision-making. We also acknowledge that our findings may not fully reflect evolving demographic trends of hormonal contraceptive use and their potential relationship with suicidal

behavior. Future research focusing on recently approved formulations, including lower-dose IUDs, will be crucial to further inform clinical practice and evidence-based guidelines.

Reviewer: 2
Dr. Catherine S Stika, Northwestern University Feinberg School of Medicine
Comments to the Author:
Both Reviewer #1 and I raised similar concerns regarding the heterogeneity of the various non-oral progestin-only contraceptives and the fact that they were lumped together in the analysis. The authors responded to our concerns with a thorough explanation, including a table showing a breakdown of both numbers of participants and their suicide risks by type of non-oral progestin-only contraceptive, which I found very informative. They also included the following statement to my question:
The findings not only validate the association of gonane-containing IUDs with suicidal behavior, but also confirm the reviewer's hypothesis that gonane-containing implants have an even greater risk increase. In contrast, pregnane-based injections were not associated with an increased risk. However, future research differentiating between these methods and exploring the distinct biological mechanisms of each method and their potential link to mood and suicidality should be performed to provide a more comprehensive understanding of their potential impact on mental health outcomes. Such analyses could provide more nuanced insights for informing clinical decision-making.
And this response to Reviewer #1:
We appreciate the reviewer's point about the heterogeneity within the 'non-oral progestin-only contraceptives' group. Combining methods like the LNG-IUD, etonogestrel implant, and DMPA injections, which have diverse mechanisms of action, limits our ability to draw specific conclusions about their individual effects on mood and suicidality. Furthermore, the dominance of the LNG-IUD group within this category adds to the challenge of isolating potential effects of other methods (Table B, below). While our findings suggest an association within this group, future research differentiating between these methods and exploring the distinct biological mechanisms of each method and their potential link to mood and suicidality is critical to provide a more comprehensive understanding of their potential impact on mental health outcomes.
However, neither the table nor any part of their response was included in their edited resubmission (Results/Discussion/Limitations or Future Research). Given that two of the three reviewers expressed concerns about this particular issue, I am surprised that the authors chose not to address it in some way within their resubmission. Because I can predict that many readers of this paper will share a similar concern, I strongly encourage the authors to reconsider their response.

Response: *We thank the reviewer for these comments and suggestions. We sincerely appreciate the insights, as well as those from Reviewers #1 and 3, which have prompted us to reflect on the implications of combining these diverse methods. In response to these concerns, we have now included a detailed breakdown of suicide risks by type of non-oral progestin-only contraceptive in Table S7. We agree that this additional information will enhance the clarity of our findings and address the heterogeneity issue more effectively. We also acknowledge the importance of differentiating between these contraceptive methods and exploring their distinct biological mechanisms, as highlighted in both this reviewer's comments and those of Reviewer #1. We agree*

that such analyses are essential for a more nuanced understanding of their potential impact on mental health outcomes, particularly concerning suicidality. Accordingly, we have now modified the manuscript text to address this issue in the Discussion.

Modified text manuscript (Discussion, page 17-18):

Additionally, while we categorized contraceptives based on their hormonal composition and administration routes, considerable variability exists within these categories, potentially influencing the interpretation of their association with mental health outcomes. Although the study was not designed and powered to examine each distinct subclass of non-oral progestin-only contraceptive, our preliminary analyses confirmed the association between gonane-containing IUDs and suicidal behavior (Table S7), suggesting that gonane-containing implants may carry an even greater risk of suicidal behavior than other non-oral progestin-only contraceptive subclasses. In contrast, compared to gonane-containing implants, pregnane-based injections appear to be associated with a relatively lower risk of suicidal behavior. Future research should focus on exploring distinct physiological mechanisms of contraceptive subclasses and their relationship with suicidality. Such studies could provide a more comprehensive perspective on mental health outcomes and yield nuanced insights to inform clinical decision-making.

Reviewer: 3
Dr. CA Hoffmire, U.S. Department of Veterans Affairs
Comments to the Author:
I appreciate the opportunity to re-review this manuscript and the authors efforts to address initial reviewer concerns. However, while some improvement was made to the manuscript, there appears to be a great deal more information in the response to reviewers document that can and should be incorporated into the manuscript itself.
Further, the authors declined to make some key changes suggested by the reviewers. The following concerns remain:

1. Two reviewers brought up notable concerns about the combined outcome. The authors did run a helpful sensitivity analysis for reviewers but chose not to include this in the manuscript itself due to concerns around sample size. Indeed, there are less than 5 suicide outcomes in the oral progestin-only group, and those findings should be suppressed due to unreliability and potential privacy concerns. However, all other groups have a sufficient number of outcomes to report reliable results. Of note this is particularly true for the non-oral progestin only group, for which the results changed (from a significant elevated risk to no association). Failing to report these results and instead stating confidence in the combined outcome is misleading. They should be added as a key sensitivity analysis and potential reasons for the observed differences warrant discussion.
***Response**: We thank the reviewer for these comments and suggestions. We sincerely appreciate the insights, as well as those from Reviewers #1 and 2, which have prompted us to reflect on the implications of combining these diverse methods. In response to these concerns, we have now included a detailed breakdown of suicide risks by type of non-oral progestin-only contraceptive in Table S7. We agree that this additional information will enhance the clarity of our findings and address the heterogeneity issue more effectively. We also acknowledge the importance of*

differentiating between these contraceptive methods and exploring their distinct biological mechanisms, as highlighted in both this reviewer's comments and those of Reviewer #1. We agree that such analyses are essential for a more nuanced understanding of their potential impact on mental health outcomes, particularly concerning suicidality. Accordingly, we have now modified the manuscript text to address this issue in the Discussion.

Table S7. Use of non-oral progestin-only contraceptives (different routes of administration) and the risk of suicidal behavior among all women in the nationwide population-based cohort, using first event in population analysis.

	N (IR)	HR* (95% CI)
No use	33,434 (4.2)	1.0
**Non-oral progestin-only (Total)**	**6,754 (5.0)**	**1.17 (1.14-1.20)**
Gonane (IUD)	4,058 (4.3)	1.15 (1.11-1.19)
Gonane (implant)	1,773 (7.3)	1.33 (1.27-1.40)
Pregnane (injection)	923 (5.3)	1.02 (0.96-1.09)

N, number of events; IR, crude incidence rate (per 1,000 person-years); HR, hazard ratio; CI, confidence interval.

* The estimates were adjusted for year of birth, attained age, civil partnership, educational level, household income, parity, history of psychiatric disorder, and additionally history of suicidal behavior in analysis of repeated events.

Modified text manuscript (Discussion, page 17-18):

Additionally, while we categorized contraceptives based on their hormonal composition and administration routes, considerable variability exists within these categories, potentially influencing the interpretation of their association with mental health outcomes. Although the study was not designed and powered to examine each distinct subclass of non-oral progestin-only contraceptive, our preliminary analyses confirmed the association between gonane-containing IUDs and suicidal behavior (Table S7), suggesting that gonane-containing implants may carry an even greater risk of suicidal behavior than other non-oral progestin-only contraceptive subclasses. In contrast, compared to gonane-containing implants, pregnane-based injections appear to be associated with a relatively lower risk of suicidal behavior. Future research should focus on exploring distinct physiological mechanisms of contraceptive subclasses and their relationship with suicidality. Such studies could provide a more comprehensive perspective on mental health outcomes and yield nuanced insights to inform clinical decision-making.

2. Reviewers added not that information on cases resulting from primary care visits is largely missing, but failed to discuss potential implications of that missing data – please add this discussion.

Response: *We thank the reviewer for pointing this out and have revised the Discussion as suggested.*

Modified text manuscript (Discussion, page 17):

Third, the limited availability of comprehensive primary care data presents a significant limitation, which may have obscured the full spectrum of mental health conditions and treatments, and impacted estimates of the reported associations, due to the risk of residual confounding.

3. A brief mention was added stating that psychiatric disorders were obtained from the patient register. Please add additional information what diagnoses/disorders were captured.

Response: *We thank the reviewer for pointing this out and have added additional information.*

Modified text manuscript (Methods, page 8):

History of psychiatric disorders (ICD F01-F99) and suicidal behavior were obtained from the National Patient Register.

4. One reviewer made an important comment about limited covariates (migraine, epilepsy). The authors responded to the reviewer but failed to add anything about this concern to the discussion as a limitation and/or other consideration – this should certainly be added.

Response: *We agree with this suggestion and have now revised the Discussion section to address this point.*

Modified text manuscript (Discussion, page 15):

Furthermore, the limited availability of covariates, especially for co-morbid conditions including migraine with aura and epilepsy, may have also influenced the findings.

5. The authors response about psychiatric covariates (i.e., regarding confounding vs. effect modification) is insufficient and not compelling or accurate. While it is certainly true that psychiatric diagnoses can be delayed and this does add complication to parsing out confounding and mediation it is very important to try to do so and widely done across the literature evaluating risk factors for suicide.

Response: *We acknowledge that our previous response did not address confounding and potential effect medication by psychiatric disorders more clearly. In the revised manuscript, we have included the description of psychiatric history in the section on confounding. We make clear that we controlled for a history of psychiatric disorders occurring before baseline and outline how this was assessed. In addition, we present stratified analyses by depression in Table S5. This addresses possible effect modification by depression, the most common psychiatric disorder, however we have insufficient power to stratify by history of other psychiatric disorders.*

Modified text manuscript (Methods, page 11):

Several additional sensitivity analyses were conducted to assess the robustness of our findings. First, to examine whether psychiatric history moderates the association between hormonal contraceptive use and suicidal behavior, we performed stratified analyses comparing women with and without a history of psychiatric disorders. Second, we conducted a focused analysis on suicide as a distinct outcome to allow for more precise interpretation. Third, we explored potential heterogeneity among non-oral progestin-only contraceptives.

VERSION 3 - REVIEW

Reviewer 1

Name **Toffol, Elena**

Affiliation **University of Helsinki Faculty of Medicine, Department of Public Health**

Date **17-Jun-2025**

COI

I thank the authors for answering the reviewers' comments. The manuscript reports now a complete overview of the analyses and results; I have a few minor suggestions and questions, mostly regarding the introduction section:

1. Introduction: I would ask the authors to double check the cited references. For example, at page 5, lines 26-28, "Large nationwide studies from Denmark reported positive associations between overall hormonal contraceptive use and the risks of suicide and depression": the cited references 13 and 14 are of a review/metanalysis article and a Swedish study, not of the Danish studies.

2. Introduction: the logical flow in the central part of second paragraph is a bit loose, in particular the sentences at lines 35-45 could be better connected to the rest of the text.

3. Introduction, the sentence at page 5, "previous studies examining the association between hormonal contraception use and mental health outcomes have primarily focused on women of younger age,15,16,18,19 which revealed strong associations between hormonal contraceptives and suicidal behavior." is imprecise. While it is true that studies 15, 18 and 19 did focus on young women, they did not focus on suicidal behavior, so the claim "which revealed strong associations between hormonal contraceptives and suicidal behavior" is incorrect. Please, reformulate more accurately.

4. Methods, page 8, lines 32-34, "History of psychiatric disorders (ICD F00-F99) and suicidal behavior were obtained from the National Patient Register." Please, mention the period covered by the "history of psychiatric disorders".

Reviewer **2**

Name **Stika, Catherine S**

Affiliation **Northwestern University Feinberg School of Medicine, Obstetrics and Gynecology**

Date **09-Jul-2025**

COI

I appreciate the ongoing efforts made by the authors to continue to improve this manuscript; however, the following comments once again reflect my perspective as a

practicing obstetrician-gynecologist and the potential implications that this paper will have on our counseling and management of all patients electing contraception.

My interpretation of the authors' newly included preliminary analysis is that nonoral pregnane-only contraception (injections) were NOT associated with an increased risk of suicide in all women: Table S7: N(IR) 923(5.3) HR 1.02(0.96 – 1.09), unlike the gonane IUD and gonane implants, which were. Why then do the authors make this statement in the Discussion, In contrast, compared to gonane-containing implants, pregnane-based injections appear to be associated with a relatively lower risk of suicidal behavior. ? It's not a "relatively lower risk of suicidal behavior"; there is no statistical increased risk. Using the phrase, "relatively lower risk" is misleading.

In addition, I am concerned that the casual reader will overlook that finding when throughout the paper the authors continue to use the descriptor, non-oral progestin-only contraception as being associated with increased suicidality. While I very much appreciate that the authors have included mention of this subsequent analysis within their paper and have added Table S7 (albeit only as a supplement), I continue to strongly encourage the authors to simply add the word, "gonane" (or another option, "in particular, gonane formulations") when they describe this association, whenever possible. This applies to both the Abstract – which is often the only part of the paper that many folk read or refer to – as well as within the Discussion.

In the Results section, given that Table S7 is available only as a supplemental table, I would encourage the authors to add to the text (2nd paragraph under the Sensitivity Analyses) that the pregnane progestin injection was not associated with increased suicide risk. Unless the reader takes the time to view the supplemental tables, they will be unaware that the authors did not observe this risk with this formulation.

In Paragraph 4 of the Discussion, the authors state, "Due to the medical risks and side effects of estrogen, many women choose hormonal contraceptives that are formulated only with progestin." In my experience, although that statement is certainly true for a relatively small set of women with medical complications, the vast majority of patients choosing contraceptive implants or progestin-releasing IUDs have done so because they are LARC options, which offer many benefits, including improved use effectiveness.

Minor comments:

1. In the Introduction the authors have newly added the statement, "Moreover, a recent Finish study reported that HC use was not significantly associated with an increased risk of attempted suicide.17 " The abbreviation, HC, is not defined prior to its use. But given that the authors never use "HC" again within the paper and instead write out "hormonal contraception", I would recommend that the authors replace HC in this sentence with hormonal contraception.

2. There are numerous uses of abbreviations within the Tables that do not have their definitions included in the legends, eg. LNG-IUD, IUD, ATC, etc.

Reviewer 3
Name Hoffmire, CA
Affiliation U.S. Department of Veterans Affairs, Rocky Mountain MIRECC for Suicide Prevention
Date 24-Jun-2025
COI

I appreciate the opportunity to continue providing reviewer feedback on this manuscript. The authors were, overall, more thorough and responsive in this revision and that is appreciated. Two key concerns remain, however:

1. In my prior review, I noted that upon first review, two reviewers brought up notable concerns about the combined outcome. However, the authors reply to this concern had nothing to do with my comment but rather addressed combined IUD types (exposure, not the outcome). I will thus repeat my concern and request for revision here. The authors did run a helpful sensitivity analysis in that first response for reviewers but chose not to include this in the manuscript itself due to concerns around sample size. Indeed, there are less than 5 suicide outcomes in the oral progestin-only group, and those findings should be suppressed due to unreliability and potential privacy concerns. However, all other groups have a sufficient number of outcomes to report reliable results. Of note this is particularly true for the non-oral progestin only group, for which the results changed (from a significant elevated risk to no association). Failing to report these results and instead stating confidence in the combined outcome is misleading. They should be added as a key sensitivity analysis and potential reasons for the observed differences warrant discussion.

2. In my prior review I also asked for the authors to clarify what psychiatric disorders were included. The authors replied that all psychiatric disorders with ICD F01-F99 were included. First, this is a category inclusive of Mental, Behavioral, and Neurodevelopmental Disorders. I am not sure that it is appropriate to include all of these – especially neurodevelopmental disorders. Second, why did the authors not categorize specific MH conditions? They are not all likely to have a uniform confounding effect.

VERSION 3 - AUTHOR RESPONSE

Reviewer: 1
Dr. Elena Toffol, University of Helsinki Faculty of Medicine

We thank Reviewer 1 for the careful reading and insightful suggestions, particularly in relation to the Introduction. We have revised the text accordingly.

Introduction: I would ask the authors to double check the cited references. For example, at page 5, lines 26-28, "Large nationwide studies from Denmark reported positive associations between overall hormonal contraceptive use and the risks of suicide and depression": the cited references 13 and 14 are of a review/metanalysis article and a Swedish study, not of the Danish studies.

Response: *We thank the reviewer for pointing this out. We have corrected the citations.*

the logical flow in the central part of second paragraph is a bit loose, in particular the sentences at lines 35-45 could be better connected to the rest of the text.

Response: *We appreciate the opportunity to improve the quality of the text. Accordingly, we have now revised this portion of the paragraph to improve the logical structure. Specifically, we restructured the sentences to better link the discussion of prior studies with the rationale for the current study.*

Modified text manuscript (Introduction, page 5):

There is currently no consensus on the potential adverse effects of hormonal contraceptive use broadly on mental health.[5–17] Several large cohort studies have found positive associations between hormonal contraceptive use and increased risks of depression and suicide,[12,13] supported by a systematic review and meta-analysis[15], and a nationally representative study reporting elevated suicidality among hormonal contraceptive users.[14] An additional cohort study among young women observed increased suicidal behavior, though this risk declined with longer duration of use.[16] In contrast, other observational studies have not demonstrated significant associations between oral contraceptive use and depression, and evidence linking hormonal contraceptive use to suicidality remains limited.[7–10] A recent large cohort study also found no significant association between hormonal contraceptive use and the risk of attempted suicide.[17]

3. Introduction, the sentence at page 5, "previous studies examining the association between hormonal contraception use and mental health outcomes have primarily focused on women of younger age,15,16,18,19 which revealed strong associations between hormonal contraceptives and suicidal behavior." is imprecise. While it is true that studies 15, 18 and 19 did focus on young women, they did not focus on suicidal behavior, so the claim "which revealed strong associations between hormonal contraceptives and suicidal behavior" is incorrect. Please, reformulate more accurately.

Response: *Thank you for your feedback. We acknowledge that the original statement conflated studies on general mental health outcomes with those on suicidal behavior. We have now reformulated the sentence to reflect that most research focuses on younger women, and that within this literature, some studies report significant associations with suicidal behavior.*

Modified text manuscript (Introduction, page 5):

Notably, the existing literature on hormonal contraception and mental health outcomes has predominantly focused on younger women,[12-16] with several studies reporting significant associations between hormonal contraceptive use and suicidal behavior within this population.[13,14,16] Given the partial overlap in risk factors for suicidality between adolescents

and adults,[18] it remains an important area of investigation whether the association between hormonal contraception and suicidal behavior persists into adulthood.

4. Methods, page 8, lines 32-34, "History of psychiatric disorders (ICD F00-F99) and suicidal behavior were obtained from the National Patient Register." Please, mention the period covered by the "history of psychiatric disorders".
Response: *We thank the reviewer for pointing this out. We have added clarification in the Methods section, specifying that the history of psychiatric disorders and suicidal behavior was obtained from clinical diagnoses recorded from 1997 onwards in the National Patient Register.*

Modified text manuscript (Methods, page 8):

Depression was considered a secondary outcome, operationally defined based on a clinical diagnosis of ICD-10 codes F32–F33 recorded in the National Patient Register, with diagnostic data available from 1997 onwards. History of psychiatric disorders (ICD F10-F99) and suicidal behavior were defined by the corresponding diagnoses anytime before the start of follow-up from the National Patient Register.

Reviewer 2:
Dr. Dr. Catherine S Stika, Northwestern University Feinberg School of Medicine

We thank Reviewer 3 for the clinically grounded insights and the constructive suggestions to improve clarity and precision in our framing of risk.

1. My interpretation of the authors' newly included preliminary analysis is that nonoral pregnane-only contraception (injections) were NOT associated with an increased risk of suicide in all women: Table S7: N(IR) 923(5.3) HR 1.02(0.96 – 1.09), unlike the gonane IUD and gonane implants, which were. Why then do the authors make this statement in the Discussion, In contrast, compared to gonane-containing implants, pregnane-based injections appear to be associated with a relatively lower risk of suicidal behavior. ? It's not a "relatively lower risk of suicidal behavior"; there is no statistical increased risk. Using the phrase, "relatively lower risk" is misleading.

Response: *Thank you for this helpful observation. We agree that the phrase "relatively lower risk" was misleading, as the analysis showed no statistically significant association between pregnane-based injections and suicide risk (HR 1.02, 95% CI 0.96–1.09). We have revised the Discussion section to clearly reflect this non-association. We appreciate the reviewer's careful attention to this point.*

Modified text manuscript (Discussion, page 18):
In contrast, pregnane-based injections were not associated with an increased risk.
2. In addition, I am concerned that the casual reader will overlook that finding when throughout the paper the authors continue to use the descriptor, non-oral progestin-only contraception as being associated with increased suicidality. While I very much appreciate that the authors have included mention of this subsequent analysis within their paper and have added Table S7 (albeit only as a supplement), I continue to strongly encourage the authors to simply add the word, "gonane" (or another option, "in particular, gonane formulations") when they describe this association, whenever possible. This applies to both the Abstract – which is

often the only part of the paper that many folk read or refer to – as well as within the Discussion.

Response: *We thank the reviewer for this thoughtful comment and fully understand the concern about clarity, particularly for readers who may rely primarily on the Abstract or overarching category labels. We agree that the distinction between gonane- and pregnane-based progestins is clinically and mechanistically meaningful and have revised the text in both the Abstract and Discussion to explicitly note that the observed association with increased suicidality is primarily driven by gonane-containing formulations, particularly IUDs and implants.*

Modified text manuscript (Abstract, page 3):

We found no overall increased risk of suicidal behavior among women using oral contraceptives or non-oral combined formulations. However, the observed increased risk associated with hormonal contraceptive use during adolescence, as well as with non-oral progestin-only contraception—particularly gonane-containing formulations—across the entire reproductive window warrants attention and further investigation.

(Discussion, page 19):

The elevated risk of suicidal behavior associated with hormonal contraceptives during adolescence and non-oral progestin-only contraception—particularly gonane-containing formulations— across the entire range of reproductive ages warrants attention and further investigation.

3. In the Results section, given that Table S7 is available only as a supplemental table, I would encourage the authors to add to the text (2nd paragraph under the Sensitivity Analyses) that the pregnane progestin injection was not associated with increased suicide risk. Unless the reader takes the time to view the supplemental tables, they will be unaware that the authors did not observe this risk with this formulation.

Response: *We thank the reviewer for the helpful suggestion. Accordingly, we have revised the Sensitivity Analyses paragraph in the Results section to clearly state that pregnane-based progestin injections were not associated with an increased risk of suicide, ensuring that this important finding is communicated directly in the main text.*

Modified text manuscript (Results, page 13):

Additional sensitivity analyses showed that the link between hormonal contraception and suicidal behavior was consistent regardless of psychiatric history (Table S5). Suicide-specific analyses were limited by sample size but did not reveal associations between hormonal contraceptive use and death by suicide (Table S6). Finally, among distinct formulations of non-oral progestin-only contraceptives, we observed an elevated risk of a first event of suicidal behavior in association with the use of gonane IUDs and implants, whereas pregnane-based progestin injections were not associated with increased risk (Table S7).

4. In Paragraph 4 of the Discussion, the authors state, "Due to the medical risks and side effects of estrogen, many women choose hormonal contraceptives that are formulated only with progestin." In my experience, although that statement is certainly true for a relatively small set of women with medical complications, the vast majority of patients choosing contraceptive implants or progestin-releasing IUDs have done so because they are LARC options, which offer many benefits, including improved use effectiveness.

Response: *We thank the reviewer for this helpful comment. We agree that the popularity of progestin-only methods, particularly IUDs, is largely due to their effectiveness and convenience as LARC options, rather than solely medical contraindications to estrogen. We have revised the Discussion to reflect this broader rationale.*

Modified text manuscript (Discussion, page 15):
While medical risks and side effects of estrogen influence some women to choose progestin-only contraceptives, the widespread use of these methods—particularly implants and IUDs—is largely driven by their effectiveness and convenience as long-acting reversible contraceptives (LARCs). Our analysis showed a consistent pattern of non-oral progestin-only contraception being more strongly associated with suicidal behavior in both adolescents and older women. Most notably, these formulations, except for pregnane-based progestins were associated with suicidal behavior across the entire reproductive lifespan.

Minor comments:
1. In the Introduction the authors have newly added the statement, "Moreover, a recent Finish study reported that HC use was not significantly associated with an increased risk of attempted suicide.17 " The abbreviation, HC, is not defined prior to its use. But given that the authors never use "HC" again within the paper and instead write out "hormonal contraception", I would recommend that the authors replace HC in this sentence with hormonal contraception.

Response: *We appreciate the opportunity to correct this and have now simply removed the abbreviation "HC" and used "hormonal contraception" in full throughout the manuscript to maintain consistency and clarity.*

2. There are numerous uses of abbreviations within the Tables that do not have their definitions included in the legends, eg. LNG-IUD, IUD, ATC, etc.

Response: *Thank you for pointing this out. We have reviewed all tables and revised the legends to ensure that all abbreviations, including LNG-IUD, IUD, ATC, and others, are now clearly defined.*

We hope that these revisions adequately address the reviewers' concerns and improve the clarity, accuracy, and utility of the manuscript. We sincerely thank the reviewers once again for their time, effort and constructive feedback, which has greatly improved the manuscript.

Reviewer 3:
Dr. CA Hoffmire, U.S. Department of Veterans Affairs

We thank Reviewer 2 for highlighting these important methodological issues. We carefully considered each point and revised the manuscript to address these concerns.

1. In my prior review, I noted that upon first review, two reviewers brought up notable concerns about the combined outcome. However, the authors reply to this concern had nothing to do with my comment but rather addressed combined IUD types (exposure, not the outcome). I will thus repeat my concern and request for revision here. The authors did run a helpful sensitivity analysis in that first response for reviewers but chose not to include this in the manuscript itself due to concerns around sample size. Indeed, there are less than 5 suicide outcomes in the oral progestin-only group, and those findings should be suppressed due to unreliability and potential privacy concerns. However, all other groups have a sufficient number of outcomes to report reliable results. Of note this is particularly true for the non-oral progestin only group, for which the results changed (from a significant elevated risk to no association). Failing to report these results and instead stating confidence in the combined outcome is misleading. They should be added as a key sensitivity analysis and potential reasons for the observed differences warrant discussion.

Response: *We thank the reviewer for this important and thoughtful comment. We share the perspective that the sensitivity analyses provide valuable context—particularly regarding the non-oral progestin-only group—and we agree that their inclusion is essential for transparency and interpretation. Accordingly, we would like to clarify that the requested sensitivity analyses were in fact already incorporated into the manuscript as Tables S6 and S7 in the prior submission. However, we mistakenly failed to refer to Table S6 in the previous response letter, and we appreciate you pointing this out.*

Table S6 presents results for suicide as a separate outcome, with suppressed reporting for groups containing fewer than 5 events to preserve privacy and prevent overinterpretation. Table S7 shows outcomes stratified by progestin subclass, revealing, for instance, the lack of association with pregnane-based injections. We have now revised the Results and Discussion sections of the manuscript to ensure these tables are clearly referenced and the implications of their findings appropriately discussed—particularly the differences in risk profiles across contraceptive subtypes and the possible mechanisms or contextual factors that may explain these discrepancies. We thank the reviewer for helping us strengthen this aspect of the manuscript.

Modified text manuscript (Discussion, page 18):

Additionally, while we categorized contraceptives based on their hormonal composition and administration routes, considerable variability exists within these categories, potentially influencing the interpretation of their association with mental health outcomes. Although the study was not designed or powered to assess each specific subclass of non-oral progestin-only contraceptives, our preliminary analyses indicated an association between gonane-containing IUDs and suicidal behavior (Table S7), suggesting that gonane-containing implants may carry an even higher risk than other non-oral formulations. In contrast, pregnane-based injections were not associated with an increased risk. A complementary sensitivity analysis focused specifically on death by suicide (Table S6) did not show a significant association with hormonal contraceptive use overall. These findings suggest that while certain formulations may be linked to suicidal behavior, including self-harm, the relationship with death by suicide remains uncertain and warrants further investigation in larger, more adequately powered studies. Future research should focus on exploring distinct physiological mechanisms of contraceptive

subclasses and their relationship with suicidality. Such studies could provide a more comprehensive perspective on mental health outcomes and yield nuanced insights to inform clinical decision-making. We also acknowledge that our findings may not fully reflect evolving demographic trends of hormonal contraceptive use and their potential relationship with suicidal behavior. Future research focusing on recently approved formulations, including lower-dose IUDs, will be crucial to further inform clinical practice and evidence-based guidelines. Finally, we note that combining different types of suicidal behaviors into a single category may have limited the ability to fully understand the nuanced relationship between hormonal contraceptive use and suicide risk.

2. In my prior review I also asked for the authors to clarify what psychiatric disorders were included. The authors replied that all psychiatric disorders with ICD F01-F99 were included. First, this is a category inclusive of Mental, Behavioral, and Neurodevelopmental Disorders. I am not sure that it is appropriate to include all of these – especially neurodevelopmental disorders. Second, why did the authors not categorize specific MH conditions? They are not all likely to have a uniform confounding effect.

Response: *We thank the reviewer for this thoughtful comment. First, we acknowledge the typographical error in our earlier response—the correct ICD range used was F10–F99, thus excluding neurocognitive disorders (F00–F09), including dementias. We have now corrected in the manuscript.*

Regarding the categorization of specific mental health conditions, our primary aim in adjusting for psychiatric history was to account for overall psychiatric morbidity as a key confounder, rather than to examine individual diagnoses. Given the broad spectrum and frequent comorbidity of psychiatric disorders, we chose a composite measure to preserve statistical power and ensure model stability across exposure groups.

That said, we fully agree that distinguishing between specific mental health conditions could provide important insights, as their associations with both hormonal contraceptive use and suicidality are unlikely to be uniform. We appreciate the reviewer's thoughtful observation and recognize this as a valuable direction for future research. We have now added it to limitation section.

Modified text manuscript (Discussion, page 18):

Future research should not only explore the physiological mechanisms of contraceptive subclasses but also consider differences across specific mental health conditions, given that their associations with hormonal contraceptive use and suicidality are unlikely to be uniform.

VERSION 4 - REVIEW

Reviewer **2**

Name **Stika, Catherine S**

Affiliation **Northwestern University Feinberg School of Medicine, Obstetrics and Gynecology**

Date **23-Sep-2025**

COI

Thank you for addressing all of my concerns. As to the suggestions I provided and the modifications made by the authors, I support publication of the current version of the manuscript.

Reviewer **3**

Name **Hoffmire, CA**

Affiliation **U.S. Department of Veterans Affairs, Rocky Mountain MIRECC for Suicide Prevention**

Date **30-Sep-2025**

COI

I appreciate that the authors have now adequately addressed all reviewer concerns.
